# Robust detection of forced warming in the presence of potentially large climate variability

**DOI:** 10.1126/sciadv.abh4429

**Published:** 2021-10-22

**Authors:** Sebastian Sippel, Nicolai Meinshausen, Enikő Székely, Erich Fischer, Angeline G. Pendergrass, Flavio Lehner, Reto Knutti

**Affiliations:** 1 Institute for Atmospheric and Climate Science, ETH Zurich, Zurich, Switzerland.; 2 Seminar for Statistics, ETH Zurich, Zurich, Switzerland.; 3 Swiss Data Science Center, ETH Zurich and EPFL, Lausanne, Switzerland.; 4 Department of Earth and Atmospheric Sciences, Cornell University, Ithaca, NY 14850, USA.; 5 Climate and Global Dynamics Laboratory, National Center for Atmospheric Research, Boulder, CO 80305, USA.

## Abstract

Climate warming is unequivocal and exceeds internal climate variability. However, estimates of the magnitude of decadal-scale variability from models and observations are uncertain, limiting determination of the fraction of warming attributable to external forcing. Here, we use statistical learning to extract a fingerprint of climate change that is robust to different model representations and magnitudes of internal variability. We find a best estimate forced warming trend of 0.8°C over the past 40 years, slightly larger than observed. It is extremely likely that at least 85% is attributable to external forcing based on the median variability across climate models. Detection remains robust even when evaluated against models with high variability and if decadal-scale variability were doubled. This work addresses a long-standing limitation in attributing warming to external forcing and opens up opportunities even in the case of large model differences in decadal-scale variability, model structural uncertainty, and limited observational records.

## Teaser

Forced climate warming can now be identified using statistical learning even under potentially large climate variability.

## INTRODUCTION

**Fig. 1.**
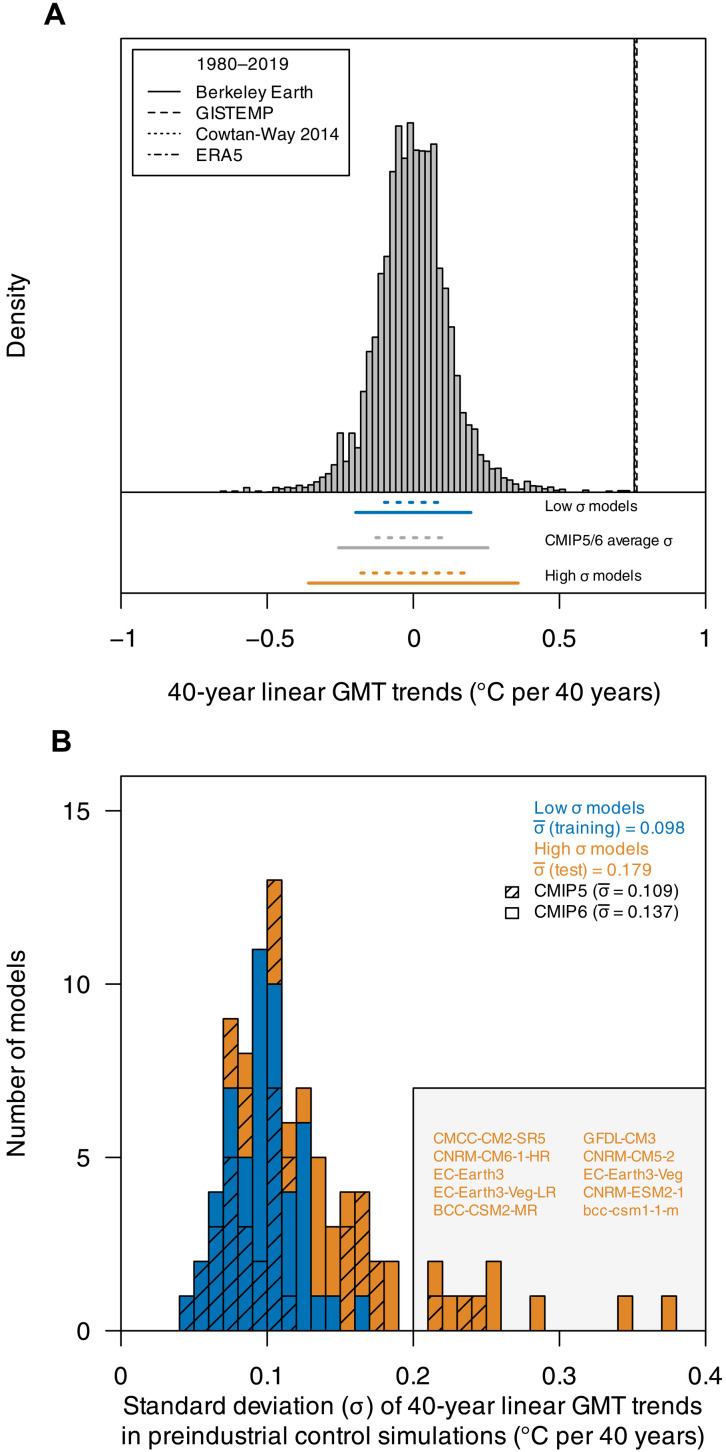
Observed warming and variability in preindustrial control simulations across climate models from the CMIP5 and CMIP6 archive. (**A**) Histogram of 40-year linear GMT trends from CMIP5 and CMIP6 preindustrial control simulations, with observed 1980–2019 trends shown as vertical black lines. Standard deviation (σ) intervals (dotted line, [−1σ,1σ]; solid line, [−2σ,2σ]) of low-variability and high-variability models are shown at the bottom of the plot. (**B**) Standard deviation of 40-year linear GMT trends separately calculated from each climate model’s preindustrial control simulation from the CMIP5 and CMIP6 archive; colors indicate low-variability and high-variability models used for training and testing, respectively, in the CMIP train-test split experiment.

The key goal of climate change detection and attribution (D&A) is to assess the causes of observed changes in the climate system (*1*). D&A ultimately aims to identify the magnitude and patterns of forced climate change in observations despite their inescapable entanglement with internal climate variability. Traditional D&A typically uses model simulated patterns (so-called fingerprints) that encapsulate the physics-based expectation of the forced climate response to individual or combined external forcings to reliably quantify the magnitude of a climate signal in observations (*2*, *3*). The probability of such a signal occurring in an unforced climate is then assessed via a systematic comparison of the strength of the fingerprint in observations and in the unforced variability of climate model preindustrial control simulations (*2*–*6*). Using variants of this approach, D&A studies have unequivocally demonstrated an imprint of externally forced climate change on multiple variables, e.g., surface and upper atmosphere temperature (*3*, *7*), the amplitude of the seasonal cycle of tropospheric temperature (*4*), humidity (*8*), and precipitation (*9*). The Intergovernmental Panel on Climate Change (IPCC)’s Fifth Assessment Report concluded that “it is virtually certain that internal variability cannot account for the observed global warming since 1951” (*1*). A recent study found that the 40-year trend in tropospheric temperature has exceeded a 5σ detection threshold (*5*). The observed 40-year global mean temperature (GMT) trend at Earth’s surface also far exceeds variability in unforced control simulations (Fig. 1A).

However, a key limitation of traditional D&A is that the robustness and estimated confidence levels depend on the ability of climate models to adequately simulate internal climate variability, particularly on longer multidecadal time scales (*1*). Comparisons between models and observations indicate that climate models show a plausible representation of global-scale temperature variability on interannual to centennial time scales (*1*, *10*–*17*), including the pattern representation of key modes of natural (internal) climate variability (*18*, *19*). Some studies infer a small role of multidecadal internal variability in the observed global temperature record (*20*–*23*) that is consistent with model simulated variability. However, other studies have raised concerns that climate models may underestimate long-range dependence and/or the magnitude of multidecadal variability at global or subglobal scales, such as in the Atlantic Ocean or Pacific Ocean (*24*–*34*).

Nonetheless, it is challenging to identify and isolate internal variability on decadal or longer time scales from observations. This is because, first, observational estimates of multidecadal climate variability are fundamentally limited by the relatively short length of the observed global record. Second, observations are, inescapably, a combination of unforced climate variability and complex forced signals (*1*). Separating these two components in observations is far from trivial and can lead to aliasing of internal variability into the estimated forced component or vice versa (*29*, *35*, *36*). Third, observations still contain potential artifacts that stem from different measurement techniques or network changes over time (*37*, *38*), particularly in the early observational record (*39*). If uncorrected, then these residual observational errors are aliased into estimates of multidecadal internal variability.

A further challenge is that model simulated patterns and magnitudes of natural variability on decadal and multidecadal time scales are highly variable across state-of-the-art climate models (*19*, *40*). For example, the standard deviation (SD) of 40-year GMT trends from preindustrial control simulations varies by up to an order of magnitude across models participating in phases 5 and 6 of the Coupled Model Intercomparison Project (CMIP) (*41*, *42*) (Fig. 1B). Moreover, the most recent climate models (CMIP6) show a larger magnitude of decadal-scale internal variability (DIV) on average than their predecessors from the CMIP5 archive, and a few CMIP6 models show high internal variability (Fig. 1B and power spectra shown in fig. S1) (*40*).

The large spread in simulated internal variability across climate models implies that the observed 40-year GMT trend of 0.76°C for 1980–2019 would exceed the SD of internally generated variability of a set of “low-variability” models by about a factor of 5 or more (Fig. 1B), corresponding to vanishingly small probabilities for the warming to be internally generated. However, the observed trend would exceed the SD of some “high-variability” models only by about a factor of 2, which would make internal variability a highly unlikely but not completely implausible explanation for a substantial fraction of the signal. Hence, model structural uncertainty in the simulation of internal variability plays a key role in D&A confidence level estimates.

Traditional optimal fingerprinting D&A studies have routinely performed residual consistency tests to check whether model simulated internal variability is consistent with the regression residuals obtained from observations (*43*). Rejection occurs if simulated internal variability estimates are deficient. Moreover, several studies have inflated simulated internal variability to account for the possibility of underestimation of model simulated variability (*9*, *44*). However, other studies showed that detection of the greenhouse gas effect on GMT remains robust irrespective of whether internal variability is stochastically represented by short-memory or long-memory processes (*45*).

Here, we test whether externally forced warming could be detected in observations if decadal and multidecadal internal variability (abbreviated henceforth as DIV) were much larger than simulated by models on average. We outline a novel D&A approach that accounts for the uncertain magnitude of DIV by explicitly including a robustness constraint in the fingerprint extraction. The constraint reduces the degree to which the uncertainty in the amplitude and patterns of global-scale DIV affects the detection metric (described in detail further below and in Materials and Methods). We evaluate the extracted D&A fingerprints and show that robustness to different representations and magnitudes of DIV is increased. Our results bolster the confidence with which externally forced trends can be detected using internal variability estimates from state-of-the-art climate models.

### Climate change fingerprints and dependence on decadal-scale internal variability

Traditional D&A fingerprints encapsulate the response of the climate system to an external forcing in a spatial or spatiotemporal pattern extracted from climate models (*2*, *3*). Subsequently, observations are typically projected onto the fingerprint and compared to the projections of model simulations of internal climate variability onto the same fingerprint, to assess whether an externally forced signal can be detected. Fingerprints may comprise a pattern of simulated forced change (*4*–*6*, *46*, *47*) or so-called optimal fingerprints, where the covariance matrix of internal climate variability is taken into account to achieve higher signal-to-noise ratios in the detection metric (*2*, *3*, *43*, *48*).

Statistical learning (or pattern recognition) can provide a complementary approach to D&A (*49*–*52*). In this framework, extraction of fingerprints from climate models can be framed as training a regression model that predicts a proxy of the forced climate response $Y_{\text{mod}}^{\text{forced}}$ (for example, a time series of the forced component of global warming derived from the ensemble average across multiple model simulations (*53*)). The regression model is based on *p* spatial predictors from a gridded field of climate variables *X*_mod_ and may be approximated linearly as

$$Y_{\text{mod}}^{\text{forced}}=X_{\text{mod}}\beta +\beta_{0}+\epsilon$$ (1)

This yields a spatial fingerprint in the form of regression coefficients (β, with intercept β_0_) that maximizes the forced signal against internal climate variability, i.e., the noise. Fingerprint extraction requires estimating a parameter that guards against overfitting (here, the ridge regression parameter λ; see Materials and Methods for all method details). An estimate of the observed forced response (${\hat{Y}}_{\text{obs}}$, with ${\hat{Y}}_{\text{obs}}=X_{\text{obs}}\hat{\beta }+\beta_{0}$) can be obtained from observations (***X***_obs_). Detection can be assessed against the null (“no forced signal”) distribution of the detection metric in unforced control simulations ($X_{\text{cntl}}\hat{\beta }+\beta_{0}$). The detection metric is hence obtained by projecting observations and unforced simulations onto the extracted fingerprint $\hat{\beta }$ (i.e., $X_{\text{obs}}\hat{\beta }$ and $X_{\text{cntl}}\hat{\beta }$), which allows comparison of the two. This approach is therefore similar to fingerprinting in traditional D&A (*2*, *3*, *43*, *48*).

However, if the climate models used to obtain variability estimates from unforced control simulations were to systematically underestimate decadal-scale variability compared to the real world, then these D&A approaches would overestimate the signal-to-noise ratio, that is, the magnitude of the forced response relative to internal variability (*1*, *3*, *17*, *54*). The result would be a bias toward earlier detection times. Accordingly, a key limitation of D&A is that “robustness of D&A of global-scale warming is subject to models correctly simulating internal variability” (highlighted in IPCC AR5 WG1 Ch. 10 (*1*); see also Fig. 1, A and B).

### Accounting for robustness to uncertain decadal-scale internal variability

Our goal is to directly address this concern by incorporating (distributional) robustness (*55*) into the statistical learning approaches described above. We account for differences in internal variability among climate models and determine whether D&A results are robust to these differences. The introduction of distributional robustness accounts for changes in the variables in Eq. 1 that are due to other, unrelated factors (in our case, DIV). If these unrelated factors were not accounted for, then they would lead to poor prediction and detection results (e.g., overestimation of confidence levels in D&A if models were to systematically underestimate DIV). Distributional robustness is achieved by extending the statistical learning optimization problem to a larger class of distributions that are relevant for our D&A setting (see also detailed discussion in Materials and Methods). Distributional robustness is related to transfer learning in machine learning (*56*) and to causal inference in statistics (*55*, *57*). Here, we seek to develop a robust regression model that successfully captures invariant properties of the climate models that show different but plausible representations of climate dynamics and variability. Hence, we extract climate change fingerprints that are robust to different climate models’ representation of DIV patterns or magnitudes. This robustness ensures that one obtains good prediction results at testing time (i.e., for climate models not used for fingerprint extraction) even for climate models with higher variability (or different spatial patterns of DIV) than in the training data.

We use anchor regression (*57*), a recently developed statistical learning technique that implements distributional robustness, to estimate the regression coefficients ($\hat{\beta }$, the “fingerprint”). Anchor regression increases robustness by protecting against variations in a specific anchor variable, which is set here to a proxy of DIV for fingerprint extraction. The global-scale DIV proxy is constructed for each climate model with at least three ensemble members by computing the difference in GMT between each ensemble member (i.e., an individual simulation) and the associated ensemble average for that specific model and subsequently low pass–filtered using a 10-year moving average. The separation of the forced, deterministic signal from random internal variability within a model ensemble via the ensemble average is widely used in the literature (*53*). This has been shown to be very effective even for ensembles with few members (*58*). However, it is not inconceivable that external forcing may modulate the behavior of internal variability, which would introduce an externally forced component in the internal variability estimated from the large ensemble. Taking the DIV proxy as the anchor variable reduces the degree to which DIV patterns project onto the anchor regression fingerprints, even in the case of large model differences in DIV. This “anchoring” ensures robustness to changes in the magnitude or patterns of internal variability. The trade-off between predictive performance and robustness is controlled by a parameter γ: Increased robustness comes at the cost of a higher prediction error in the unchanged case, i.e., if the test models’ variability strongly resembled the training models’ variability (see Materials and Methods for all details on fingerprint extraction and anchor regression and fig. S4 for a schematic illustration of the method).

### Testing potentially high internal climate variability in our detection framework

We evaluate detection results based on anchor regression against three other detection metrics. These detection metrics are (i) GMT, a key climate change metric used in policy assessments; (ii) a detection metric that is based on the mean warming pattern (MWP) across models but without optimization against internal variability [following (*5*) and earlier D&A literature (*2*–*4*)]; and (iii) a detection metric that maximizes the signal against the noise of internal variability to predict the forced response, using ridge regression, but without robustness constraints (i.e., the ridge regression fingerprint) (*51*).

We evaluate D&A results for two different experiments (as described below) by considering the implications of a potential systematic model underestimation of the amplitude of observed decadal variability. Each experiment imposes differences in the magnitude of internal variability between the climate models used for fingerprint extraction (“training models”) and the models used for the evaluation of D&A results (“test models”).

#### CMIP train-test split (experiment 1)

We split the CMIP5 and CMIP6 archive into a set of “low-variability models” for fingerprint extraction (i.e., training models) and a set of “high-variability models” (i.e., test models) for evaluating D&A estimates. The partitioning of models is based on each model’s magnitude of DIV, which is estimated for each model from preindustrial control simulations (see fig. S2 for an illustration; training and test models in historical and scenario simulations and in preindustrial control simulations are listed in tables S1 and S2, respectively). Test models have higher variability not only on decadal time scales but also on multidecadal to centennial time scales (spectra shown in fig. S2E). Training and test models both show that regional variability in a few key regions is associated with global-scale DIV, such as in the North Atlantic, the East Pacific, or at high latitudes (*40*), but high variability models show a stronger functional relationship with global DIV (fig. S3).

#### Artificial DIV scaling (experiment 2)

We artificially change the SD of the main modes of DIV in each model’s preindustrial control simulation by a scaling factor *s* (*s* ∈ [0.5,2,3]). The main modes of DIV correspond here to the first 10 empirical orthogonal functions (EOFs) of decadally smoothed control simulations, and we scale the SDs, i.e., the square root of the respective eigenvalues (variances). In this “artificial DIV scaling” experiment, fingerprints are extracted from the original model simulations, but then the observed forced response is evaluated against the scaled variability estimates from preindustrial control simulations.

Both experiments address hypothetical situations in which state-of-the-art climate models underestimate the true but uncertain DIV of the real world and thus enable us to test the possible implications for D&A estimates. Estimates of 40-year forced warming trends are evaluated on the basis of each fingerprint and exclusively compared to climate models that have not been used for extracting the fingerprints (see Materials and Methods).

## RESULTS AND DISCUSSION

### Illustration of D&A based on DIV anchor

**Fig. 2.**
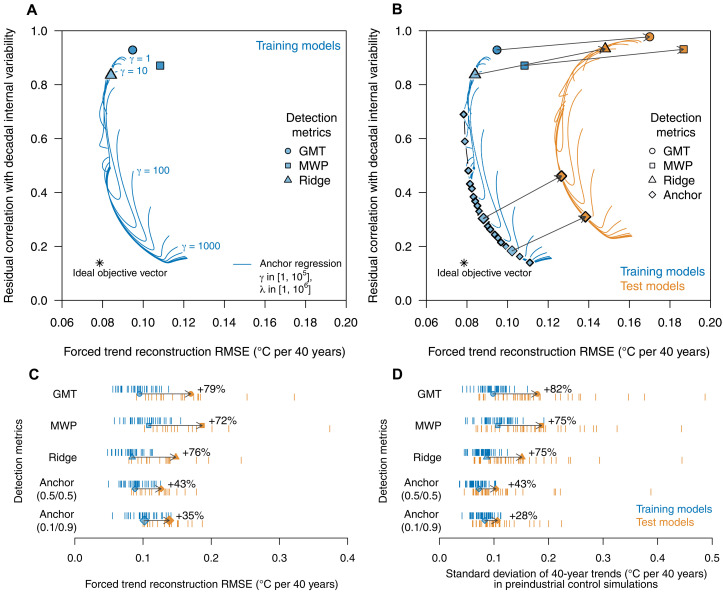
Illustration of the trade-off between predictive accuracy and robustness to decadal-scale variability in low-variability (training) models and high-variability (test) models in the CMIP train-test split experiment. (**A** and **B**) RMSE for the prediction of 40-year linear trends in forced temperature, evaluated using different climate change detection metrics (GMT, MWP, and optimized ridge regression detection metric), and the degree to which DIV projects onto these fingerprints [i.e., correlation of prediction residuals ($\hat{Y}-Y$) with DIV] for (A) low-variability training models and for (B) high-variability test models in the CMIP train-test split experiment. Anchor regression estimates for different hyperparameter values (anchor hyperparameter γ ∈ [1,10^5^], ridge regularization hyperparameter λ ∈ [1,10^6^]) are shown as blue (training models) and orange (test models) lines. Blue diamonds along anchor regression lines indicate Pareto optimal estimates, i.e., illustrating the trade-off between reducing RMSE and reducing the residual correlation with DIV. The two larger blue diamonds indicate the anchor (0.5/0.5) and anchor (0.1/0.9) detection metrics used in the paper. (**C**) RMSE calculated from 40-year trends in historical simulations. (**D**) Standard deviation (SD) of 40-year trends in preindustrial control simulations for training (blue) and test (orange) models for all detection metrics in the CMIP train-test split experiment. Black arrows show the average change in (C) RMSE and (D) SD of 40-year trends for between low-variability training models and previously unseen high-variability test models.

We start by illustrating the trade-off between prediction performance and robustness to DIV in the “CMIP train-test split” (Fig. 2A). Prediction performance in the reconstruction of forced warming is evaluated using the root mean squared error (RMSE; Fig. 2A, *x* axis) between 40-year linear trends in each detection metric (in each historical ensemble member) and the forced warming trend. RMSE is computed separately for each climate model and then averaged across the set of training and test models. The “true” forced response is taken from a smoothed ensemble average for all models that have at least three ensemble members (see Materials and Methods for all details). We quantify robustness to DIV as the correlation of the residuals from the prediction (${\hat{Y}}^{\text{forced}}-Y^{\text{forced}}$, converted to 40-year trends) with DIV (i.e., the corresponding 40-year linear trend in the anchor variable), which reflects the degree to which patterns of DIV project onto the respective fingerprints.

Traditional detection metrics such as GMT or the MWP-based detection metric show relatively small errors in the reconstruction of the 40-year forced warming trend across training models (Fig. 2A and fig. S5). However, prediction residuals correlate strongly with DIV (Fig. 2A), which would imply that D&A results would be overconfident if DIV was systematically underestimated by current climate models. Detection metrics based on anchor regression fingerprints achieve similar, and for some parameter values even reduced, prediction errors across a wide range of parameter values (γ, λ; blue lines in Fig. 2A). However, these anchor regression–based detection metrics also notably reduce the correlation of the residuals with DIV, which implies that DIV does not project as strongly onto these fingerprints (Fig. 2A and fig. S6 for individual model results), thus indicating increased robustness to DIV. The trade-off between performance and robustness is visible on the lower left corner in Fig. 2A, where a Pareto optimal front emerges, that is, an increase in robustness (reduction in residual correlation with DIV) comes at the cost of an increase in prediction error and vice versa.

Next, we evaluate the performance of detection metrics across the high-variability test models (Fig. 2B). We select two anchor parameter combinations (γ, λ) along the Pareto optimal solutions (*59*) that jointly minimize a weighted combination of residual correlation with DIV and prediction error among the training models (Fig. 2A). These are chosen to reflect (i) an equal weighting of RMSE versus residual correlation (anchor parameters, 0.5/0.5) and (ii) a preference for a reduction in residual correlation over RMSE reduction (anchor parameters, 0.1/0.9; for details see Materials and Methods), thus favoring robustness.

The prediction error increases substantially for high-variability test models (Fig. 2B). For example, RMSE increases by about 72 to 79% for traditional detection metrics [Fig. 2, B (black arrows) and C]. Conversely, the RMSE for detection based on anchor regression increases only by about 35 to 43% (Fig. 2C). Moreover, the magnitude of internal variability in the detection metric in the absence of a forced change, that is, for preindustrial control simulations projected on the respective fingerprints, shows a strong increase for traditional detection metrics (Fig. 2D, 75 to 82% increase in the SD of 40-year trends). Anchor regression–based detection metrics show a smaller increase of 28 to 43% (Fig. 2D), hence again indicating increased robustness. The anchor regression detection metrics also considerably reduce the spread in the magnitude of unforced variability across all climate models (Fig. 2D and fig. S7), and model-specific unforced variability estimates are more consistent even when the annual global mean SD is scaled to be the same in all models (fig. S8).

Overall, this illustrative example shows that anchor regression detection metrics are more robust under the distributional change imposed by the high-variability test models, thus achieving the lowest prediction error in the CMIP train-test split scenario (Fig. 2C). In addition, anchor regression estimates increase robustness (thus reducing spread) across climate models’ representations of patterns and magnitude of unforced variability estimates (Fig. 2D).

### Understanding robustness to DIV

**Fig. 3.**
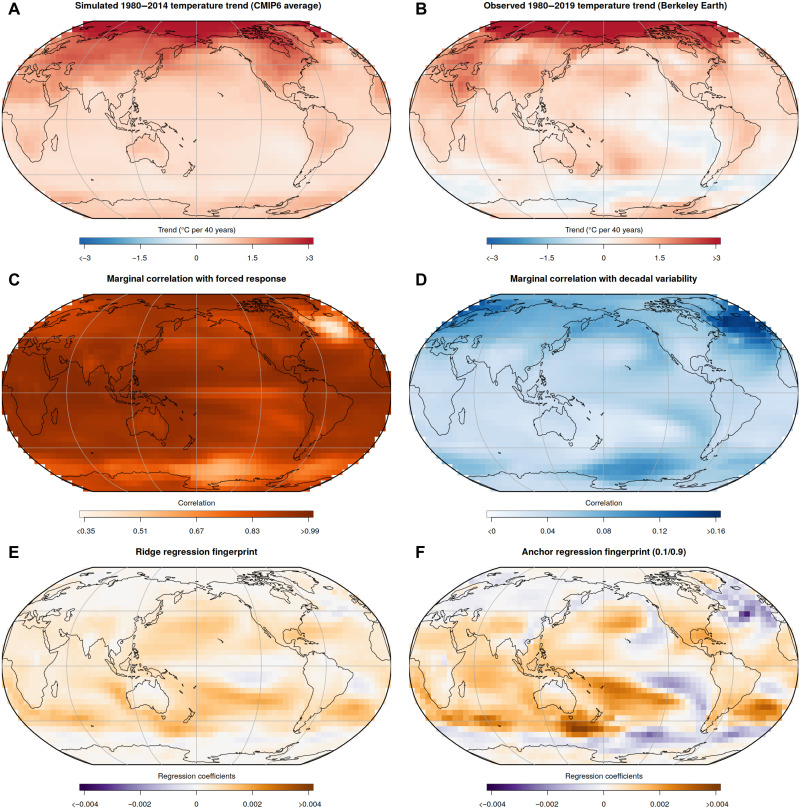
Patterns of simulated and observed temperature trends, local correlations with forced response and decadal-scale variability, and ridge and anchor regression fingerprints. (**A**) Average of historical 1980–2014 temperature trends across climate model simulations from the CMIP6 archive. (**B**) Observed linear 1980–2019 temperature trends from the Berkeley Earth temperature dataset (*75*). (**C** and **D**) Correlation of each grid cells’ local annual temperature with global forced temperature response (C) and DIV (D) (“marginal correlation”). (**E** and **F**) Fingerprints of estimated ridge regression (E) (λ = 22,909) and anchor regression (F) (λ = 40,738, γ = 500) coefficients for the prediction of the forced response. Anchor regression fingerprint is shown illustratively for the Pareto optimal solution with preference weighting *w*_RMSE_ = 0.1 and *w*_resCorr_ = 0.9 (Anchor 0.1/0.9).

To understand the link between the anchor fingerprint and robustness to DIV, we investigate temperature trends and key patterns of variability. Simulated multimodel historical CMIP6 temperature trends (1980–2014) show warming with strong Arctic amplification (*60*) and a pronounced land-sea warming contrast (*61*) (Fig. 3A). More moderate warming trends are simulated in the North Atlantic (*62*) and the Southern Ocean (*63*) (Fig. 3A).

Observed 1980–2019 temperature trends display key features of the simulated forced warming pattern, such as strong Arctic amplification, a moderate land-sea warming contrast, and a warming hole in the North Atlantic (Fig. 3B). However, observed temperature trends disagree with the multimodel forced pattern in some oceanic regions where observations have shown nonuniform warming, or even cooling, such as a horseshoe-shaped region in the Pacific Ocean and extensive cooling in the Southern Ocean. In the Pacific, these differences are consistent with internal variability related to the Interdecadal Pacific Oscillation (*64*, *65*). In the Southern Ocean, cooling trends have been attributed to natural multidecadal variability related to Southern Ocean convection (*66*) in tandem with delayed forced warming (*63*).

Across the models’ historical and future scenario simulations, most regions are highly correlated with the global forced response, except for the North Atlantic, the Southern Ocean, and the equatorial Pacific (Fig. 3C and see fig. S10 for results from individual models), indicating that these regions show substantial variability unrelated to long-term warming. Conversely, regional correlations with DIV are relatively weak across the CMIP archive (Fig. 3D). However, several regions show stronger associations with DIV, including, in particular, the Eastern Pacific, the Southern Ocean, and the North Atlantic (Fig. 3D and see fig. S11 for individual models’ control simulations). Although models differ substantially in their magnitude of simulated internal variability both globally (Fig. 1B) and regionally (e.g., fig. S3), some key features of DIV patterns are relatively robust across the majority of climate models (fig. S11). These include, for example, the association of global DIV with regional variability in the East Pacific, North Atlantic, and at high latitudes (*40*).

Climate change D&A has long made use of the distinct pattern differences between the more globally coherent forced pattern and the smaller-scale patterns of internal variability (*3*, *54*). It is against this background that climate change fingerprints can be interpreted and understood. The traditional MWP fingerprint (*5*), which is not optimized against the noise of internal variability, directly reflects the multimodel MWP (Fig. 3A and MWP fingerprint shown in fig. S9). The MWP fingerprint thus shows larger warming over the Arctic and over continental land areas, consistent with the CMIP6 trends shown in Fig. 3A. The ridge regression fingerprint (Fig. 3E), which optimizes the signal against the noise of internal variability, consists of mostly positive regression coefficients in several oceanic regions. The fingerprint shows smaller regression coefficients over the Arctic, in continental regions, and in the tropical Pacific and Southern Ocean. These are regions with large natural variability unrelated to the forced warming and thus with lower signal-to-noise ratios (*49*, *51*).

The anchor regression fingerprint (Fig. 3F shows Anchor 0.1/0.9, and Anchor 0.5/0.5 and uncertainties are shown in fig. S9) shares similarities with the ridge regression fingerprint in that oceanic regions generally receive larger regression coefficients than continental land regions and the Arctic, with mostly positive coefficients in the Western Pacific Ocean and the Indian Ocean. However, the anchor fingerprint assigns negative coefficients in regions that are strongly associated with DIV (cf. Fig. 3, D and F), particularly the North Atlantic, the Eastern Pacific (North Pacific), and the Southern Ocean. These negative coefficients reduce the degree to which patterns of DIV project onto the anchor fingerprint. Increased robustness to DIV in anchor regression estimates (as seen in Fig. 2, A to D) can thus be understood as counterbalancing DIV in a few key regions of variability via negative coefficients while still ensuring a good prediction of forced warming at the global scale. Moreover, it is remarkable that the anchor regression fingerprint identifies three key regions (Eastern Pacific, Southern Ocean, and North Atlantic) that are also associated with large decadal-scale variability in observations and have experienced muted or reduced warming trends in 1980–2019 (cf. Fig. 3, B and F; no observations are used in the fingerprint extraction).

### Detection of forced warming under scaled DIV

**Fig. 4.**
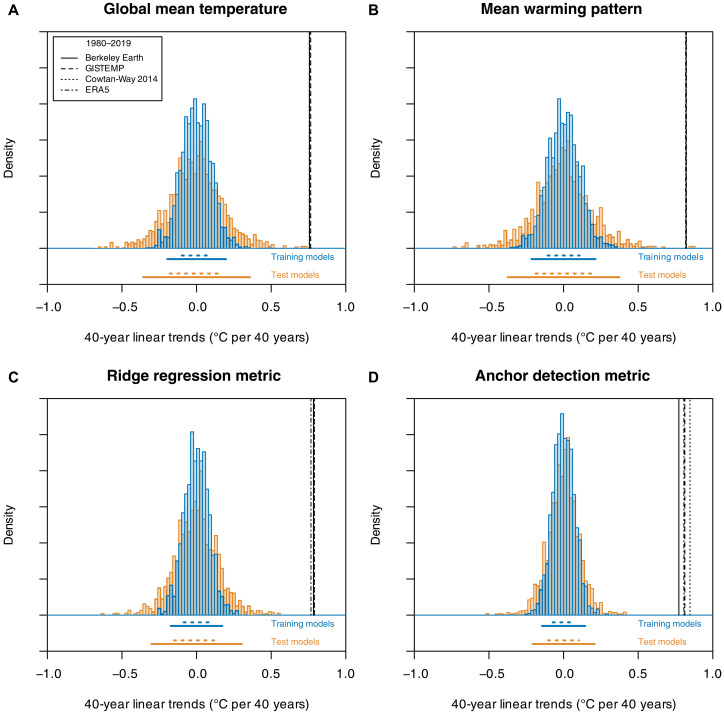
Detection of observed 40-year temperature trend (1980–2019) in the presence of potentially large decadal-scale internal variability in the CMIP train-test split experiment. Histogram of 40-year trends from preindustrial control simulations of the low-variability training models and high-variability test models for the (**A**) GMT, (**B**) MWP, (**C**) ridge regression, and (**D**) anchor regression (Anchor 0.5/0.5) detection metrics, shown alongside observed and reanalysis-based 1980–2019 trends (black vertical lines). The dotted and solid horizontal lines below each panel indicate [−1σ,1σ] and [−2σ,2σ] ranges, respectively, for the set of training and test models.

Detection is shown in Fig. 4 for 40-year trends in GMT and the anchor regression detection metric for the CMIP train-test split experiment. Observed GMT has increased by about 0.76°C over the past 40 years (1980–2019). Our best estimate of global forced warming based on the anchor regression (0.5/0.5) detection metric is 0.8°C per 40 years with a range of 0.77° to 0.85°C per 40 years across observational datasets (Fig. 4; best estimates for recent 40-year forced warming for the MWP and ridge regression are 0.82° and 0.79°C, respectively). The difference between global mean warming and forced warming as diagnosed by the anchor detection metric is thus rather small and may be due to the partly offsetting effect of cooling induced by the phasing of multidecadal modes of internal variability.

However, estimates of the SD of 40-year GMT trends in an unforced climate increase substantially between the set of low-variability training models [${\bar{\sigma }}_{\text{train}}=0.098{}^{\circ}$C (40 years)^−1^] and the high-variability test models [${\bar{\sigma }}_{\text{test}}=0.179{}^{\circ}$C (40 years)^−1^], corresponding to an 82% increase. Similar sensitivity of natural variability estimates in the CMIP train-test split experiment is observed for the traditional MWP fingerprint (75%) and the ridge regression fingerprint (75%; Fig. 2D), shown in Fig. 4. Conversely, the anchor regression detection metric yields a substantially smaller increase in variability between training and test models [+43% and +28% in the SD for the Anchor 0.5/0.5 and Anchor 0.1/0.9 metrics, respectively, with ${\bar{\sigma }}_{\text{train}}=0.072{}^{\circ}$C (40 years)^−1^, ${\bar{\sigma }}_{\text{test}}=0.104{}^{\circ}$C (40 years)^−1^ for Anchor 0.5/0.5], resulting overall in a more robust detection (Fig. 4D).

**Fig. 5.**
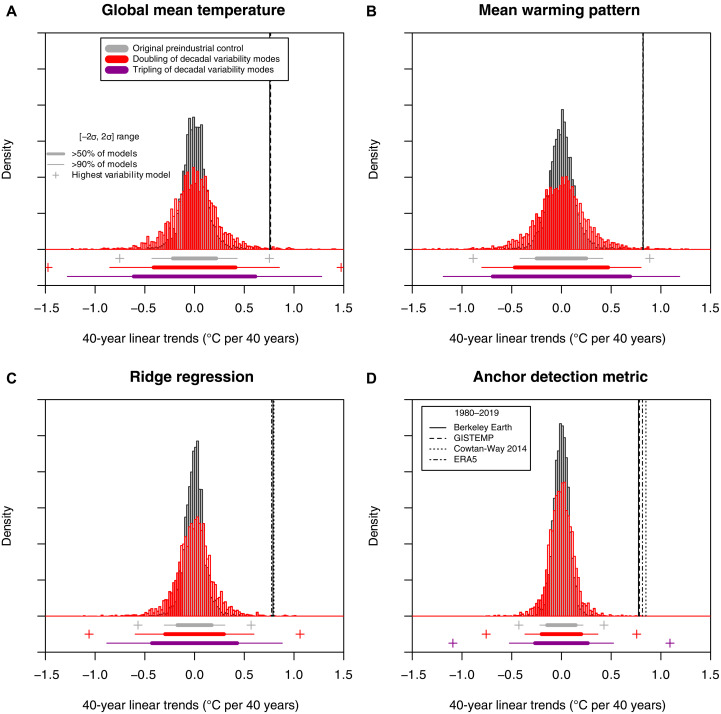
Detection of observed 40-year temperature trend (1980–2019) in the presence of potentially large decadal-scale internal variability under doubling and tripling of the standard deviation of the 10 dominant decadal modes of variability. Histogram of 40-year trends from preindustrial control simulations for the (**A**) GMT, (**B**) MWP, (**C**) ridge regression, and (**D**) anchor regression (Anchor 0.5/0.5) detection metrics, shown alongside observed and reanalyzed 1980–2019 trends (black vertical lines).

Next, we focus on the artificial DIV scaling experiment to address the question of whether detection results would remain valid if decadal-scale modes of internal variability were doubled or tripled. Doubling the SD of the main modes of DIV causes effectively a near-doubling of the SD of 40-year GMT trends in preindustrial control simulations (+87.8%; Fig. 5A and fig. S12 for individual models) and for the MWP fingerprint. The observed trend slope would still exceed a 2σ detection threshold, i.e., higher than extremely likely according to IPCC terminology (*67*), if tested against a “median-variability” CMIP model even for doubling or tripling of the SD of internal variability (Fig. 5A). However, if tested against high-variability CMIP models (e.g., >90% of models in Fig. 5A), the detection of externally forced warming would not exceed a 2σ threshold under doubling of internal variability modes for GMT. The sensitivity to DIV doubling is similarly high for the MWP and ridge regression detection metrics (Fig. 5).

The anchor regression detection metric derived from unforced control simulations is more robust to scaling of internal modes of variability (Fig. 5D and figs. S13 and S14 for individual models). Detection of externally forced warming would exceed a 2σ detection threshold under a doubling of the main internal variability modes even for the model that shows the largest magnitude of internal decadal-scale variability in the CMIP archive (Fig. 5D) or even under a tripling of variability for at least 90% of all individual models. Detection is also robust if the SD of internal variability (not only the 10 main DIV modes as defined above) is doubled or tripled (fig. S13). Detection results, including the increased robustness to uncertain variability estimates provided by the anchor regression methodology, are consistent across different trend time scales with typically higher fractions of attributable warming for longer time scales (fig. S15). The anchor regression methodology is particularly effective on relatively short analysis time scales, such as for D&A of 30- and 40-year trends, compared to the traditional detection metrics (fig. S15).

However, a few limitations of the detection method introduced here need to be discussed. First, anchor regression fingerprints are trained to protect against variations in a specific anchor variable. Here, DIV is the selected anchor. This implies that if variables other than the anchor variable change or if models collectively misrepresent patterns of variability, then anchor regression detection results would not necessarily increase robustness compared to traditional metrics (unless one could specifically protect against these aspects). However, climate model evaluation against observations does not indicate a fundamental misrepresentation of the patterns of major modes of climate variability (*11*, *19*). Second, anchor regression requires the selection of the robustness hyperparameter γ. The magnitude of distributional changes up to which anchor regression estimates can provide robustness thus depends on the parameter choice (*57*). The method therefore implies a trade-off between robustness and prediction performance but, as seen here, may even outperform more traditional detection methods under a relatively high level of robustness (see Fig. 2). Third, it should be noted that we assess the detection of temperature responses to external forcing throughout this paper, which, by definition, includes anthropogenic and natural forcings. Natural solar forcing has decreased over the past few decades (*1*), and the vertical fingerprints of natural forcings have been shown to be inconsistent with the observed warming (*68*). Therefore, it can be assumed that the external forcing over recent decades is dominated by anthropogenic forcing, consistent with IPCC assessments (*1*).

In summary, our results show that externally forced warming over the past 40 years is detected in observations even under two hypothetical scenarios of high DIV: (i) a train-test split of the CMIP archive, where detection is assessed against a subset of high-variability models; and (ii) a doubling of the SD of decadal-scale modes of variability that yields a robust detection result even if tested against any CMIP model individually.

### The minimum fraction of warming attributable to external forcing

Detection results presented so far in this paper addressed the basic detection test of whether internal variability alone could have caused the full observed warming trend. By testing against each individual CMIP model’s unforced DIV estimates, we now estimate for each detection metric the minimum fraction of the observed 40-year trend that cannot be explained by internal variability (i.e., must be externally forced) at an extremely likely level [>95% (*67*), corresponding to >1.65σ in a one-sided Gaussian distribution; models used listed in table S2] or, in other words, the largest plausible contribution of internal variability to warming.

**Fig. 6.**
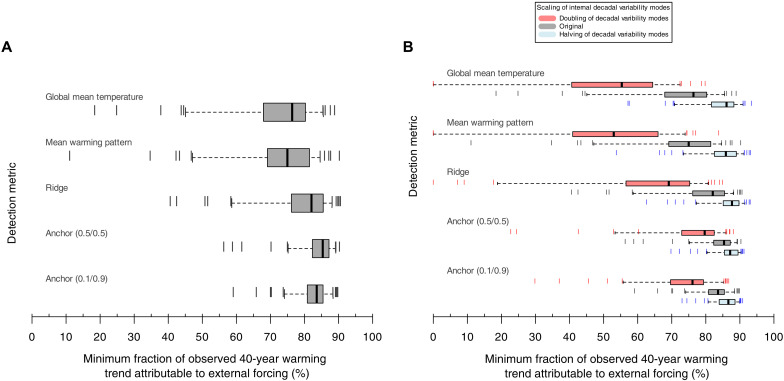
Detection of forced warming under potentially high decadal-scale internal variability against each individual model’s preindustrial control simulation. (**A**) Box plots show the minimum fraction of the observed 40-year trend (1980–2019) that internal variability cannot account for (that is, caused by external factors) at the extremely likely level (*67*), >95% probability in a one-sided Gaussian distribution, tested separately against each model’s preindustrial variability estimates and shown for different detection metrics (GMT, MWP, ridge, and anchor detection metrics). Boxes show interquartile range across models, and whiskers show the 5th and 95th quantile across the distribution of models. (**B**) As in (A) but including the model distribution for doubling (×2) and halving (×0.5) the main modes of unforced decadal-scale variability.

For a median-variability CMIP model, at least 85% of warming over the past four decades is attributable to external forcing for the anchor regression (0.5/0.5) detection metric at the extremely likely level (Fig. 6A, box plot medians) and at least 76% for the GMT and MWP detection metrics. However, for the CMIP models that show the highest internal variability, traditional GMT and MWP detection metrics show minimum fractions of 40-year observed warming trends attributable to external forcing that are even below 50% for several models and as low as 10% for the highest-variability model (Fig. 6A). Model disagreement and structural uncertainty may thus hamper detection statements. For the anchor detection metrics, however, even the highest-variability model shows a fraction of attributable warming for the 40-year trend of at least about 56% (Fig. 6A), and the minimum fraction of attributable warming falls below 70% for only three climate models.

Anchor regression yields more robust detection estimates across the diverse representation of internal variability in individual climate models, thus reducing model structural uncertainty in D&A. While the magnitude of DIV differs substantially across climate models, the anchor regression method protects against model variability differences in key spatial regions (such as the East Pacific, the North Atlantic, or the Southern Ocean; Fig. 3) associated with potentially large climate variability. The method thus makes use of the distinct differences between the DIV patterns (which have large amplitudes and large intermodel differences in these key regions) and the more globally coherent forced warming pattern. Forced versus internal pattern differences are well known in the literature (*3*, *4*, *40*, *68*) and have long been used in traditional climate change D&A (*3*, *54*). The results are therefore more consistent across models even when GMT variability is scaled to be the same in all models’ preindustrial control simulations (fig. S8), suggesting that the anchor-based detection method is less sensitive to differences in model feedbacks and the resulting spatial patterns. The overall fraction attributed to external factors is higher than in traditional detection metrics, i.e., the anchor method improves both detectability and robustness at the same time.

When either doubling or halving the SD of the main modes of DIV, the effect of the anchor fingerprint becomes even more clear (Fig. 6B): The anchor regression detection metric shows substantially smaller changes (in either direction) as compared to the traditional detection metrics for all models but especially in the high-variability tail of the distribution of climate models. In particular, even if the main natural decadal variability modes were doubled, at least 55% of the observed 40-year trend cannot be explained by internal variability in all but five models at the extremely likely level for the anchor detection metric (Fig. 6B). The median across CMIP models shows that about 75% of the observed 40-year trend could be attributed to external forcing under a doubling of the SD of the main DIV modes using the anchor regression detection metric (Fig. 6B). If the SD of internal variability were scaled up by a factor of 2 (instead of doubling only the SD of the 10 main modes of DIV), then about 70% of the observed 40-year warming trend could be attributed to external forcing for the median CMIP model, and externally forced warming would still be detected in the highest variability CMIP models (fig. S14). One would need to quadruple the SD of internal variability for GMT in the median CMIP model to be able to reject the detection of externally forced warming, and for the anchor detection metric, the SD of internal variability would have to be scaled up by more than a factor of 6 for the median CMIP model.

### Conclusion

We introduced an approach to climate change D&A that explicitly increases robustness to the large structural uncertainty in magnitude and patterns of DIV across state-of-the-art climate models (*40*). Concerns have been raised that some climate models may underestimate variability, and potential model errors in internal variability are a key remaining limitation in D&A (*1*).

Here, we demonstrated that a novel approach from statistical learning increases the robustness of D&A to different representations of DIV. The D&A methodology relies on anchor regression (*57*) to extract a fingerprint (in the form of a set of regression coefficients) that encapsulates the expectation of the forced signal against internal variability but under a specific robustness constraint linked to DIV (i.e., the anchor variable). With this approach, externally forced global warming over the past 40 years can be detected with high confidence in observations even with those climate models that simulate the largest magnitude of decadal-scale variability.

We find a global forced temperature trend of 0.77° to 0.85°C over the past 40 years [based on three observational temperature datasets and the anchor regression (0.5/0.5) fingerprint], which is slightly higher than the observed GMT warming trend of 0.76°C per 40 years (1980–2019). It is extremely likely that at least 85% of the warming trend extracted by the anchor regression fingerprint over the past 40 years (based on the median across CMIP models) and 56% based on the highest-variability model cannot be explained by internal variability. Even if the SD in preindustrial control simulations were doubled, about 70% of the observed 40-year warming trend could be attributed to external forcing for the median variability CMIP model, and detection would remain robust even for the highest-variability CMIP model. The spread in variability estimates across climate models is substantially reduced as well, and hence, the sensitivity of D&A results to uncertainties related to DIV across different climate models is reduced.

The present work addresses a long-standing limitation of D&A (*1*) and opens previously unidentified avenues to increasing robustness of D&A in the presence of poorly quantified or uncertain, yet important features linked to the magnitude and patterns of DIV and model structural uncertainty. Anchor regression can detect externally forced patterns even on time scales of only three decades and under high variability (see fig. S15). Therefore, anchor regression may help to address more intricate D&A problems in climate variables with limited spatial or temporal coverage or large internal variability, such as in the water cycle. In this context, anchor regression D&A could take into account multiple climate variables simultaneously, thus improving signal identification. Future work may also aim to diagnose multidecadal internal variability on continental or global scales while anchoring against externally forced components or to diagnose specific climate forcings (e.g., anthropogenic aerosols), while anchoring against possible variations in other forcings (e.g., greenhouse gases or natural forcings).

## MATERIALS AND METHODS

### Climate change detection method

Traditional D&A is based on “fingerprints” (*2*, *3*), which are typically extracted from climate model simulations and represent patterns of the climate system response to external forcing (*1*, *5*). Fingerprints are typically stored in the form of a spatial or spatiotemporal pattern and may be rotated in low-noise directions to achieve better signal-to-noise characteristics (*2*, *3*). In traditional D&A, observations and unforced control simulations are projected onto these simulated fingerprint patterns. This yields a one-dimensional test statistic that reflects the degree to which observations and control simulations show similarity to the fingerprint pattern. Trends in the observed test statistic are compared to the distribution of trends in the test statistic from preindustrial control simulations to assess whether externally forced climate signals in observations can be detected against the noise of internal variability (*5*).

Here, complementary to traditional approaches, we frame D&A as a regularized linear regression model that relates patterns of simulated climate variables to a one-dimensional proxy of forced climate change, *Y*_mod_ ∈ ℝ*^n^*, (*50*, *51*) through a set of regression coefficients (β ∈ ℝ*^p^*; see Eq. 1)

$$Y_{\text{mod}}^{\text{forced}}=X_{\text{mod}}\beta +\beta_{0}+\epsilon$$ (2)

Here, the *n* × *p* matrix *X*_mod_ represents a collection of spatial patterns of annual temperatures (here, *p* = 2592 spatial grid cells derived from a 5° by 5° regular longitude-latitude grid), with *n* representing the number of available samples for fingerprint extraction (i.e., the number of model years across the CMIP5 and CMIP6 archives; see “Data processing” section for details on the simulations used). *Y*_mod_ is a vector of length *n* that represents the global average forced temperature response for each of the *n* samples, and β_0_ and ϵ represent the intercept and the error term, respectively. Details on how we obtain our forced response proxy *Y*_mod_ from climate model simulations are given below. All input data are centered and standardized before model fitting; therefore, the intercept (β_0_) is omitted in the description of ridge and anchor regression below.

Our detection method is closely linked to traditional fingerprinting as described above (*2*, *3*, *5*): The set of regression coefficients (β *∈ ℝ^p^*, a column vector of length *p*) from the linear regressionmodel in Eq. 1 projects the high-dimensional annual temperature maps from models or observations into a one-dimensional detection space (specified by the target of the prediction in Eq. 1, i.e., reflecting a global-scale forced response proxy) that is then used as the test statistic to assess whether we can detect the influence of external forcing. Thus, the set of regression coefficients (in the form of a map shown in Fig. 3, E and F) can be interpreted in a similar way to a fingerprint in traditional D&A: The regression coefficients encapsulate the signal of the forced response but optimized against internal variability such as to best predict the one-dimensional forced response proxy of interest $Y_{\text{mod}}^{\text{forced}}$. Because the number of predictors *p* is large and the grid cell predictors are highly correlated, we use a ridge regression regularization to estimate and constrain regression coefficients (see “Ridge regression” section).

In a second step, after the regression coefficients $\hat{\beta }$ have been estimated, observations (*X*_obs_) and unforced climate model control simulations (*X*_cntl_) are projected onto the fingerprint

$${\hat{Y}}_{\text{obs}}=X_{\text{obs}}\hat{\beta }+\beta_{0}$$ (3)

$${\hat{Y}}_{\text{cntl}}=X_{\text{cntl}}\hat{\beta }+\beta_{0}$$ (4)

A detection test can be carried out by assessing whether the observed estimate of the detection metric (${\hat{Y}}_{\text{obs}}$), or trends derived from this metric, falls within the distribution of unforced control simulations (*51*). This testis again closely connected to traditional D&A in which trend signals are assessed against the noise of unforced climate simulations (*3*,*5*), as described above. Here, we calculate linear 40-year trend slopes from our annual estimates of forced change (${\hat{Y}}_{\text{obs}}$) and for unforced control (${\hat{Y}}_{\text{cntl}}$) and transient (${\hat{Y}}_{\text{mod}*}$) model simulations (where _mod*_ indicates that those simulations have not been used in fingerprint extraction) to compute different detection metrics and to assess detection in observations. All model estimates shown in the evaluation or analysis of detection results are based on fingerprints extracted from a different set of climate models, that is, detection is tested exclusively on the basis of previously unseen models.

Overall, the anchor detection method used here can be seen conceptually as linked to and complementary to traditional D&A that uses model simulated fingerprints (or “guess patterns”) to assess whether external forcing can be detected in observations against the noise of internal variability (*1*, *3*, *5*). In the method outlined here, fingerprints are stored in the form of regression coefficients and optimized via regularized linear regression (Eq. 1, regularization described below) to achieve better signal-to-noise characteristics. In contrast to the widely used algorithm of optimal fingerprinting (*1*, *43*), however, our method does not attempt to model the high-dimensional spatiotemporal structure of observations in a forward way. Instead, climate model simulations and observations are projected into a one-dimensional detection space (given by Eq. 1, that is reflecting a global-scale forced response proxy) to test whether one can detect the presence of external forcing.

### Statistical learning techniques

A key issue is that the number of predictors is large (here, *p* = 2592) and the predictors are highly correlated, which could lead to overfitting (*69*) of the linear regression model. In our climate application, overfitting would result in a noisy, nonsmooth, unconstrained, and thus unphysical fingerprint. To extract regression coefficients, we make use of regularized linear regression models (ridge regression) and a statistical learning technique that includes a robustness constraint on DIV (anchor regression) in addition to the smoothness regularization provided by ridge regression.

### Ridge regression

Ridge regression is a standard technique to deal with a large number of correlated predictors by penalizing model complexity, thus avoiding overfitting through shrinkage of regression coefficients (known as “regularization”) (*69*). Consider a linear model as specified by Eq. 1, where the goal is to minimize a loss function given by the residual sum of squares (RSS)

$$\text{RSS}=\sum_{i=1}^{n}{\left(y_{i}-x_{i}^{T}\beta \right)}^{2}={\left||Y-X\beta |\right|}_{2}^{2}$$ (5)

The subscript *i* indicates the value for each given year (of the total number of years from all simulations). To avoid large regression coefficients, a ridge regression penalty based on the 𝓁_2_-norm, i.e., the sum of squared coefficients, is then added to the objective function such that

$$\hat{\beta }=\underset{\beta }{\text{argmin}}[\text{RSS}+\lambda {\left||\beta |\right|}_{2}^{2}]$$ (6)

The regularization parameter λ (also called a hyperparameter) determines the amount of shrinkage and hence balances the bias-variance trade-off of the ridge regression model (see the “Fingerprint extraction and evaluation of predictions” section for details on the selection of the hyperparameter λ). The ridge regression model yields small but nonzero regression coefficients, and the coefficients are smoothly distributed among correlated predictors (*69*). That is, we extract a fingerprint that is smooth in space, thus capturing the spatial correlation inherent to climate variables.

Regularizing statistical models using a penalty term (e.g., the 𝓁_2_-norm, corresponding to ridge regression, also called Tikhonov regularization) is a standard and popular approach and has been used in numerous climate applications, for instance, to regularize neural network weights to extract indicator patterns of the forced response (*49*). A previous detection study targeting individual time steps implemented and described the method outlined here (*51*). A standard optimal fingerprinting algorithm also uses regularization to estimate the covariance matrix (*48*).

### Distributional robustness

In a regression setting as described above, extraction of fingerprints ($\hat{\beta }$) is performed by minimizing a population loss 𝓁, typically squared error loss, over a distribution *P* that spans one or multiple climate models

$$\hat{\beta }=\underset{\beta }{\text{argmin}}\;E_{(X,Y)∼P}[\ell (Y,X\beta )]$$ (7)

However, regression models fitted on a training dataset are not per se robust in a hypothetical setting when the regression model is applied if external factors change, i.e., if the regression model is applied in a different so-called environment (*57*). For example, in the D&A context, one may think of a different environment if the models used for fingerprint extraction would systematically underestimate decadal-scale variability as compared to some test climate models or the real world.

Ideally, the estimated regression coefficients should provide robust predictions under reasonable distributional changes, i.e., if some external factors or environments change, which motivates distributional robustness as a key concept in statistical learning (*55*). That is, our goal is to minimize prediction error not only for a certain population distribution *P* as in Eq. 7 but also for a broader class of distributions 𝒬 (*55*)

$$\hat{\beta }=\underset{\beta }{\text{argmin}}\;\underset{Q\in Q}{\text{sup}}E_{(X,Y)∼Q}[\ell (Y,X\beta )]$$ (8)

The optimization problem above can be interpreted as protecting against a worst-case scenario among a reasonable class of distributions 𝒬 that contains *P*. Good prediction results are hence achieved even for “reasonable” distributional changes, but distributional robustness comes at the cost of a somewhat higher prediction error if the test distribution closely resembles the training distribution. Distributional robustness is related to causal inference, since causal regression models also display invariant properties across different environments or domains of application (*55*).

### Anchor regression

Anchor regression is a statistical learning technique that implements the concept of distributional robustness with respect to distributional changes in a given anchor variable (*55*, *57*, *70*). Anchor regression has been used in previous work to protect against the influence of specific forcings in a D&A context (*50*).

An illustration of the intuition behind anchor regression in the context of our climate change detection method is shown in fig. S4: Because decadal and multidecadal variabilities affect annual temperature patterns and long-term trends, DIV may project onto fingerprints that seek to capture the external forcing signal (e.g., fig. S4A), adversely affecting D&A results. This is seen, for instance, through the high positive correlation between the prediction residuals (${\hat{Y}}^{\text{forced}}-Y^{\text{forced}}$) and DIV (Fig. 2, A and B), especially for simple detection metrics such as GMT, MWP, or in the ridge regression detection metric. Because DIV may therefore influence D&A statements, we aim to reduce this dependency. Ideally, prediction residuals would be uncorrelated with (or orthogonal to) DIV. Intuitively, this orthogonality would then provide more robust D&A estimates, even if climate models used to extract fingerprints underestimated internal variability (e.g., Fig. 2, A and B). The rationale behind anchor regression is to use the variation in the anchor variable (here, DIV) during training of the models to reduce the correlation between the anchor variable and prediction residuals (fig. S4, B and C), which would increase the robustness of the obtained statistical model (e.g., fingerprints of the forced response) to hypothetically increased variability in the anchor variable (i.e., larger DIV) in the test setting.

Throughout this paper, we use DIV as the anchor variable *A* ∈ *ℝ^n^* (see below for details about how we extract *A* from climate models for training). Hence, we aim for a robust climate change detection procedure in the context of substantial uncertainty in the representation of DIV in climate models. In observations, DIV is likewise very uncertain and challenging to quantify because observations are simultaneously affected by internal variability and forced changes. Because our statistical model is trained on climate model DIV information (where DIV, corresponding to the anchor variable *A*, can be reliably estimated) to be robust to model DIV differences, we do not need to estimate DIV in observations: The fingerprint already contains the robustness information from training.

Anchor regression coefficients (${\hat{\beta }}^{\gamma }$) are estimated by

$${\hat{\beta }}^{\gamma }=\underset{\beta }{\text{argmin}}\underset{\left(a\right)}{\underbrace{{\left||(I_{n}-\Pi_{A})(Y-X\text{β})|\right|}_{2}^{2}}}+\underset{\left(b\right)}{\underbrace{\gamma {\left||\Pi_{A}(Y-X\text{β})|\right|}_{2}^{2}}}$$ (9)

where *I_n_* ∈ ℝ^*n*×*n*^ is the identity matrix and Π*_A_* ∈ ℝ^*n*×*n*^ is the matrix that projects onto the column space of *A*, given by Π*_A_* ≔ *A*(*A^T^A*)^−1^*A^T^* (*57*).

The first term (a) in Eq. 9 aims to minimize empirical error in the training distribution (disregarding variations in *A*), and the second term (b) increases robustness to changes (or shifts) in *A* with the degree of robustness given by the anchor regression parameter γ (sometimes called “causal” regularization parameter) (*70*). For γ = 1, anchor regression coincides with the ordinary least squares solution (*57*).

Because the robustness or causal regularization with respect to the anchor variable does not protect against overfitting in a general sense (i.e., the large number of predictors *p*), we include a ridge regression penalty based on the 𝓁^2^-norm ($\lambda {\left\|\beta \right\|}_{2}^{2}$ as in Eq. 6), and the anchor regression estimator becomes

$${{\hat{\beta }}^{\gamma ,\lambda }=\underset{\beta }{\text{argmin}}||(I_{n}-\Pi_{A})(Y-X\beta )||{}_{2}^{2}+\gamma ||\Pi_{A}(Y-X\beta )||}_{2}^{2}+\lambda {\left||\beta |\right|}_{2}^{2}$$ (10)

Including the ridge regression penalty shrinks regression coefficients and hence constrains the overall complexity of the model, thus ensuring that the maps of regression coefficients (fingerprints, ${\hat{\beta }}^{\gamma ,\lambda }$) are relatively smooth inspace. Our anchor regression estimate thus depends on two regularization hyperparameters (the anchor regression parameter γ and the ridge regression parameter λ). The selection of the two hyperparameters follows a multiobjective optimization strategy, that is, the optimal values of λ and γ are chosen from the Pareto curves in Fig. 2A (indicated by blue diamonds in Fig. 2A; see detailed description of the multiobjective optimization strategy below). For fixed (γ, λ), Eq. 10 can be solved in a straightforward way on a transformed dataset (*57*), using standard ridge regression techniques (*50*)

$${\hat{\beta }}^{\gamma ,\lambda }=\underset{\beta }{\text{argmin}}{\left||\tilde{Y}-\tilde{X}\beta |\right|}_{2}^{2}+\lambda {\left||\beta |\right|}_{2}^{2}$$ (11)

where $\tilde{X}=(I_{n}-\Pi_{A})X+\sqrt{\gamma }\Pi_{A}X$ and $\tilde{Y}=(I_{n}-\Pi_{A})Y+\sqrt{\gamma }\Pi_{A}Y$ contain the transformed input and output data values.

### Fingerprint extraction and evaluation of predictions

We estimate regression coefficients and select the hyperparameters for ridge regression and anchor regression (λ, γ) via a resampling strategy, where the set of climate models available for training is successively split into a set of models used to learn the regression coefficients (i.e., “model fitting”) and a set of models used for validation. That is, for *B* = 50 iterations, we randomly split training samples from *k* climate models (*k* = 14 for CMIP-split experiment and *k* = 19 for training on all models) successively into 50% training data (resulting in *k*/2 climate models, with *n* = 2000 randomly subsampled annual data points for each of the *k*/2 climate models; subsampling is performed to ensure every model receives the same weight in the optimization) and 50% validation data (from the other half of climate models). We solve for the anchor regression coefficient estimates ${\hat{\beta }}_{b}^{\gamma ,\lambda }$ with *b* = 1, …, *B* on the training data for a sequence of 100 candidate λ values in a logarithmically spaced sequence (λ ∈ [1; 10^6^]) and 19 candidate γ values (γ ∈ [1; 10^5^]). The coefficients (${\hat{\beta }}_{b}^{\gamma ,\lambda }$) are used to calculate estimates/predictions of the forced response ${\hat{Y}}_{\text{mod}}$ for the validation models. Hence, different combinations of models are used for training and validation over each of the 50 iterations, and each model ends up about 50% of the time in the training and validation set. Ridge regression and anchor regression coefficients are estimated on the basis of annual data from the CMIP5 and CMIP6 archive using historical (1870–2005 in CMIP5 and 1850–2014 in CMIP6) and Representative Concentration Pathway (RCP) scenarios (2006–2100 in CMIP5 and 2015–2100 in CMIP6) simulations to ensure a large training record (see the “Data processing” section for all details regarding prior data processing). For each (λ, γ) combination, we compute error estimates (see the next paragraph) for each climate model’s forced response estimates (${\hat{Y}}_{k}$), averaged over alliterations in which the respective model was used as a validation model.

We evaluate the performance and robustness of our predictions based on the historical climate model simulations (Fig. 2, A and B): To assess the prediction error in the reconstruction of forced warming, we compute the RMSE between 40-year linear trends in each detection metric (and in each historical ensemble member) and the forced warming trend. RMSE is thus calculated as $\text{RMSE}=\sqrt{\sum_{i=1}^{n}{\left(y_{40\;\text{years},i}-{\hat{y}}_{40\;\text{years},i}\right)}^{2}}$, where *n* is the total number of 40-year trends from all simulations and *i* is the value for each given 40-year trend.

RMSE is calculated separately for each climate model and subsequently averaged for the set of training models and test models shown in Fig. 2 (A and B). The true forced response is the smoothed ensemble average of the respective climate model (only models with at least three ensemble members are considered). Furthermore, we evaluate the robustness (i.e., the degree to which patterns of DIV project onto the respective fingerprints) via calculating the correlation of 40-year trends in prediction residuals (${\hat{Y}}^{\text{forced}}-Y^{\text{forced}}$) with DIV (i.e., the corresponding 40-year linear trend in the anchor variable *A*). We calculate these error estimates from 40-year linear trends across the CMIP archive from historical simulations only and for trends starting every 10 years (i.e., 1860–1899, 1870–1909, ..., 1970–2009).

Note that the residuals are calculated as “predicted minus observed” (i.e., $\hat{Y}-Y$) for ease of interpretation in Fig. 3A (i.e., a correlation in cases where internal variability projects onto the fingerprints), instead of “observed minus predicted” that would be a more standard definition of residuals. Note that the test models shown in Fig. 2 are not used for training (fingerprint extraction). The final regression coefficient estimates (fingerprints) shown in Fig. 3 are averaged coefficients over the *B* = 50 iterations, i.e., ${\hat{\beta }}^{\gamma ,\lambda }=\frac{1}{B}\sum_{b=1}^{B}{\hat{\beta }}_{b}^{\gamma ,\lambda }$), which is equivalent to predicting using all the 50 individual fingerprints (from the 50 resampling sets of models, each generating different fingerprints) and averaging the predictions. The main advantage of the validation strategy used here is to stabilize coefficient estimates in a relatively high-dimensional setting (i.e., a relatively high number of predictors *p*). An overview of the variability of the fingerprints (maps of coefficients) across the model resampling subsets used for fingerprint extraction is shown in fig. S9.

### Selection of regularization parameters

Validation model error estimates are used to select the hyperparameters for ridge regression (λ) and anchor regression (γ, λ). Note that ridge regression can be seen as a special case of anchor regression (as defined in Eq. 10) with γ = 1.

For ridge regression coefficient estimates (${\hat{\beta }}^{\lambda }$), we select the hyperparameter λ to retain the most regularized model (largest λ value) within 5% of the minimum RMSE averaged over all validation models. Results are practically unchanged if λ is selected within a reasonable range around the minimum RMSE (Fig. 2A).

For anchor regression coefficient estimates (${\hat{\beta }}^{\gamma ,\lambda }$), the selection of regularization parameters is more difficult and depends on the application (*70*), because we are dealing with two hyperparameters that each address different objectives. The anchor regression parameter (γ) encourages distributional robustness with respect to *A*, and the ridge regression parameter (λ) avoids overfitting through coefficient shrinkage.

To illustrate the trade-off between the two objectives, we show one diagnostic related to each objective: (i) RMSE from the prediction and (ii) the residual correlation with the anchor for robustness in Fig. 2A. In this context, parameter selection can be seen as a multiobjective optimization problem (*59*), where we select parameters to jointly minimize both metrics in Fig. 2A. Blue lines illustrate the two diagnostics for all (γ, λ) combinations for the validation models within the training models. The trade-off between the two objectives is clearly visible in that the minimization of both metrics simultaneously is unachievable, as, in this case, a decrease in one metric is unavoidably associated with an increase in the other. A Pareto optimal front emerges where none of the objectives can be improved without deteriorating the other objective (*59*), i.e., toward the lower left corner in Fig. 2A, and parameter selection thus ultimately depends on individual preferences of weighting a decrease in one metric against an increase in the other. The lowest individually achievable values for each metric (unachievable in practice) are shown as the “ideal objective vector” ($z_{i}^{+}$) in Fig. 2A (the opposite vector that captures the worst values for each metrics individually along the Pareto front is known as the “nadir objective vector,” $z_{i}^{\text{nad}}$). The hyperparameters are then selected as follows.

First, we introduce a set of positive weights *w_i_* ≥ 0, *i* ∈ {1,2} that determine our preference for a decrease in residual correlation versus a decrease in RMSE along the Pareto front, where the index *i* runs over the two objectives (residual correlation and RMSE). The weights are chosen to add up such that *w*_resCorr_ + *w*_RMSE_ = 1, where *w*_resCorr_ and *w*_RMSE_ encapsulate the two objectives. In Fig. 2A (blue diamonds), we illustrate Pareto optimal solution for weights in the range *w*_RMSE_ ∈ [0,0.05,0.1, …,1] and the corresponding *w*_resCorr_ ∈ [1,0.95,0.9, …,0]. Results of anchor regression estimates are shown and discussed in the paper for two weight combinations: (i) *w*_RMSE_ = 0.5 and *w*_resCorr_ = 0.5, denoted “Anchor 0.5/0.5,” and (ii) *w*_RMSE_ = 0.1 and *w*_resCorr_ = 0.9, denoted “Anchor 0.1/0.9.” Second, we select the parameter combination (γ, λ) that produces a solution that fulfils the criterion [p. 97 in (*59*)]

$$\text{minimize}\;\underset{i=1,2}{\text{max}}[w_{i}\frac{\left|f_{i}(\gamma ,\lambda )-z_{i}^{+}\right|}{z_{i}^{\text{nad}}-z_{i}^{+}}]$$ (12)

across all available (γ, λ) combinations. The denominator in Eq. 12 ($z_{i}^{\text{nad}}-z_{i}^{+}$) achieves a rescaling of the two objectives *f_i_*(γ, λ) to the same magnitude using the ranges spanned by the ideal and nadir objective vectors [following p. 18 in (*59*)], such that the two objective functions are normalized, i.e., take values in the range [0,1].

That is, we calculate for all (γ, λ) combinations and a given set of weights (*w*_RMSE_, *w*_resCorr_), the weighted normalized distance between each of the two objectives with their ideal values, and retain the maximum value across the two objectives. Next, we select the (γ, λ) combination that produces the lowest value across all combinations. This type of parameter selection is a standard weighted global criterion method in multiobjective optimization [“Tchebycheff problem,” p. 68 and p. 98 in (*59*)].

### Traditional detection metrics

We compare our detection metric based on anchor regression to three other detection metrics. These include (i) a GMT metric, (ii) a detection metric based on the MWP (MWP fingerprint), and (iii) a detection metric based on ridge regression, a special case of anchor regression as discussed above for γ = 1.

### Global mean temperature

GMT is widely used in policy discussions around climate change and has been the basis for nonpattern-based detection studies (*71*). GMT trends can be thought of as a detection metric resulting from a projection of temperature patterns onto a simple area-proportional D&A fingerprint (*3*).

### MWP fingerprint

A widely used traditional detection metric is based on a fingerprint that encapsulates the pattern of forced warming. We extract the MWP fingerprint in a very similar way to previous studies (*4*, *5*, *7*, *46*).

First, we average over each individual model’s historical and RCP8.5 (in CMIP5; or historical and SSP585 in CMIP6) simulations, where multiple simulations are available (separately for each year and every grid cell *x* located on the 5° by 5° grid). Second, we average across all CMIP5 (or CMIP6) models to arrive at a multimodel average surface air temperature change, $\overset{̿}{S}(x,t)$ [following notation in (*5*)], as a function of location *x* and time *t*. The first EOF of appropriately area-weighted anomalies ${\overset{̿}{S}}_{\text{anom}}(x,t)$ encapsulates the MWP across the CMIP5 and CMIP6 archives and is referred to here as the MWP fingerprint (shown in fig. S9). In traditional D&A, observations and preindustrial control simulations are projected onto the MWP fingerprint to estimate signal-to-noise ratios either using a Euclidean metric (nonoptimized standard regression) or an inverse noise covariance matrix (detection based on optimal fingerprinting) (*3*–*5*, *7*, *46*). Here, we regress the forced response across the CMIP archive on the nonoptimized MWP detection metric (obtained from projecting model simulations onto the MWP fingerprint), to estimate the forced response. Thus, the MWP fingerprint allows us to predict the forced response based on the expected mean warming signal only (but without any optimization against internal variability). The MWP fingerprint yields consistent and reliable estimates of the forced response across the CMIP archive (fig. S5).

### Data processing

For the extraction of fingerprints (training of statistical learning methods), we first select all model simulations from the CMIP5 and CMIP6 archive from historical and scenario simulations (RCP2.6, RCP4.5, and RCP8.5 in CMIP5; SSP1-1.9, SSP1-2.6, SSP2-4.5, SSP3-7.0, SSP4-3.4, and SSP5-8.5 in CMIP6) that contain at least three different ensemble members (see tables S1 and S2 for a detailed overview of model simulations and ensemble members). Different model versions from the same modeling center are treated as different models in our analysis.

Second, the data are preprocessed as follows: For each model, all data are regridded to a regular 5° by 5° grid, and annual surface air temperature values are converted for each model to anomalies relative to a common 1870–1920 reference period. Because individual ensemble members from the same model show, in some cases, offsets against each other in their long-term averages (likely because they branched off from the control run at different times), we adjust the long-term average (1870–2005) of each ensemble member to the long-term average of all ensemble members of that respective model.

Third, we extract the GMT forced response through averaging over all available ensemble members in each model. This procedure is a standard way of evaluating the forced response in large ensembles, because internal variability that arises randomly in each realization (ensemble member) averages out if the ensemble size is large enough (*53*). As noted above, we only consider models with at least three ensemble members (see table S1) and additionally smooth the forced response estimates using a locally weighted scatterplot smoothing procedure (*72*) (smoothing parameter is chosen as 0.75 combined with a second-order polynomial smoothing). Smoothing time series to reduce internal variations and to extract estimates of forced responses is a standard procedure (*73*). However, smoothing alone would be likely to conflate multidecadal internal variability with the forced response (*74*); therefore, multiple ensemble members for each model and scenario are required to separate the forced response from DIV (see, e.g., fig. S2 for an illustration of the separation).

Next, we compute an estimate of DIV, which is used as the anchor variable (*A*) for the fingerprint extraction. Spread between ensemble members within a specific model is thought of conceptually as internal variability superimposed upon the (common) forced response (*53*) (but there may also be interactions between the forced response and internal variability). Here, we estimate the contribution of internal variability for any ensemble member and at any time step *t* on a global scale by subtracting the forced response *Y*^forced^ from the annual GMT time series. Then, we obtain an estimate of DIV by smoothing using a 10-year running mean filter. Figure S2 illustrates the procedure for estimating the forced response *Y*^forced^ and DIV for two climate models: MPI-ESM1-2-LR (fig. S2) shows a relatively small DIV magnitude, while EC-Earth3 (fig. S2B) is among the models that produce the largest DIV magnitude. Note that the magnitude of DIV extracted from historical and scenario simulations as described above correlates strongly, across all CMIP models, with DIV estimates extracted from preindustrial control simulations in the absence of a forced response (Pearson correlation of *R* = 0.85; fig. S2F). This confirms that separation of DIV from the forced response in scenario simulations is robust.

Subsequently, fingerprints are extracted (see the “Fingerprint extraction and evaluation of predictions” section above) from historical (1870–2005 in CMIP5 and 1850–2014 in CMIP6) and scenario simulations (2006–2100 in CMIP5 and 2015–2100 in CMIP6). Scenario simulations were included to realize a training record that is as large as possible for the statistical learning algorithm and to extract a fingerprint that represents the global-scale forced response under different forcing conditions (all used models and scenarios are listed in table S1). The reconstruction of the forced response based on the extracted fingerprints (shown in Fig. 2, A and B) is evaluated on the basis of historical simulations only. Last, we project preindustrial control simulations onto the fingerprints, according to Eq. 4. Preindustrial control simulations are linearly detrended to remove any potential drift, and the first 100 years of each model’s control run are discarded. Next, linear 40-year trends in the detection metrics are calculated from control run segments starting every 10 years in each control run. Trends are shown as a pooled distribution of unforced variability in Figs. 4 and 5 (but with equal weights for each model) and as a distribution of model-specific internal variability in Fig. 6. Models are grouped by model family (e.g., the MPI “family”; see table S1) for fingerprint extraction, fingerprint evaluation in Fig. 3, and for deriving the model-specific equal weights for the histograms in Figs. 4 and 5. In contrast, the analysis of model-specific internal variability in Fig. 6 is based on the preindustrial control simulations of in total 91 individual model variants (e.g., MPI-ESM-LR from the MPI family).

Detection statements are made on the basis of the observed 40-year trend compared to the SD of 40-year trends in preindustrial control simulations. We assess the quality of the observations to be good, the range of uncertainty sampled by the models to be appropriate and the robustness of the method to be high. We therefore interpret the statistical confidence that results from the analysis based on an extremely likely threshold following IPCC uncertainty language [>95% (*67*); corresponding to >1.65σ in a one-sided Gaussian distribution].

### Experiments with scaled internal climate variability

In the “CMIP train-test split (experiment 1)” section, we split the CMIP5 and CMIP6 models in two sets of low-variability and high-variability models based on the DIV values in their preindustrial control simulations (illustrated in fig. S2). Low-variability models are used for fingerprint extraction, and high-variability models are used to assess detection. Note that if any model variant shows high variability (e.g., NorESM2-LM), all other variants of the same model family are also selected as high-variability models (e.g., NorESM1-M) to ensure that all model variants of the same model family are either in the training or testing set. Consequently, not all high-variability model variants (shown in Fig. 1B) exceed the magnitude of DIV of all low-variability models. However, high-variability models exceed the magnitude of DIV in low-variability models (SD of decadal global temperature anomalies) by a factor of 2 (fig. S2C), and power spectra of high-variability models also show much higher variability on multidecadal time scales (fig. S2E). Results for the CMIP train-test split are shown in Figs. 1 to 4. Additional analysis of patterns of variability in low-variability and high-variability models is shown in fig. S3.

In the “Artificial DIV scaling (experiment 2)” section, we first smooth each CMIP model’s preindustrial control simulation with a decadal running average and then calculate EOFs from the smoothed data. We refer to these subsequently as“decadal EOFs.” We scale the square root of the 10 largest eigenvalues by a scaling factor *s* (*s* ∈ [0.5,2,3]) and transform the data back to its original coordinates (that is, *s* = 1 corresponds to the original preindustrial simulations). The distributional change thus corresponds to a scenario where climate models would underestimate for *s* > 1 (or overestimate for *s* < 1), the magnitude of the 10 first modes of DIV. Results of the scaling of the main modes of DIV are shown in Figs. 5 and 6. In addition to experiment 2, we scale the SD of preindustrial control simulations directly (i.e., scaling all modes of variability instead of only the first 10 EOFs of DIV); and respective results are shown in figs. S13 and S14.

### Observationally based datasets and reanalyses

Observationally based datasets are used to calculate 40-year GMT trends in Fig. 1A, in the spatial trend analysis in Fig. 3B, and the different detection metrics in Figs. 4 to 6. Three gridded temperature datasets are used with near-global spatial coverage over the past four decades: (i) the Berkeley Earth Surface Temperature dataset (BEST) (*75*), (ii) the Cowtan and Way temperature reconstruction (CW14) (*76*), which is based on HadCRUT4 (*77*), and (iii) the National Aeronautics and Space Administration’s GISS Surface Temperature Analysis version 4 (GISTEMP-v4) (*78*). All three datasets are bilinearly regridded to the same regular 5° by 5° grid used for the analysis of the climate models. All three datasets achieve global or near-global coverage via a statistical reconstruction and infilling of observational gaps using station-based land 2-m temperatures blended with sea surface temperature measurements. Sea surface temperatures show slightly less warming than air temperatures above the sea (*76*), which may imply small differences between forced response estimates derived from observations (based on sea surface temperatures) and climate models (based on surface air temperatures). Observed estimates are hence likely somewhat conservative. To assess the impact on our detection metrics arising from blending of sea surface temperatures and land 2-m temperatures in observational datasets, we used both (i) blended data (skin temperature over the ocean combined with surface air temperature over land) and (ii) nonblended data (surface air temperatures) from two different reanalysis products: ERA5 (*79*) and version 3 of the Twentieth Century Reanalysis (*80*). Differences between detection metrics based on blended and nonblended data are very small for ERA5. Differences in the Twentieth Century Reanalysis are slightly larger, but 40-year trends based on blended data are smaller, i.e., detection based on blended data is conservative (fig. S16). Detection statistics in Figs. 4 and 5 are based on 40-year trends calculated from each individual observational dataset (1980–2019). The statistics presented in Fig. 6 are based on the average 40-year observed trend across all four datasets. Although we have shown that our detection results are robust to differences in these four statistically infilled observational datasets, our future work will further explore detection results and associated uncertainties if the entire analysis is restricted to the coverage of observations.
