# Supplementary material for: Robust detection of forced warming in the presence of potentially large climate variability
Source: Sci Adv. 2021 Oct 22;7(43):eabh4429. doi: 10.1126/sciadv.abh4429 (PMC8535853; doi:10.1126/sciadv.abh4429)
Supplement: Supplementary file 1 — Figs. S1 to S16 Tables S1 to S2 [file sciadv.abh4429_sm.pdf]

Science Advances
AAAS

Supplementary Materials for

Robust detection of forced warming in the presence of potentially large climate variability

Sebastian Sippel*, Nicolai Meinshausen, Enikő Székely, Erich Fischer,
Angeline G. Pendergrass, Flavio Lehner, Reto Knutti

*Corresponding author. Email: sebastian.sippel@env.ethz.ch.

Published 22 October 2021, *Sci. Adv.* **7**, eabh4429 (2021)
DOI: 10.1126/sciadv.abh4429

This PDF file includes:

Figs. S1 to S16
Tables S1 to S2

Supplementary Figures

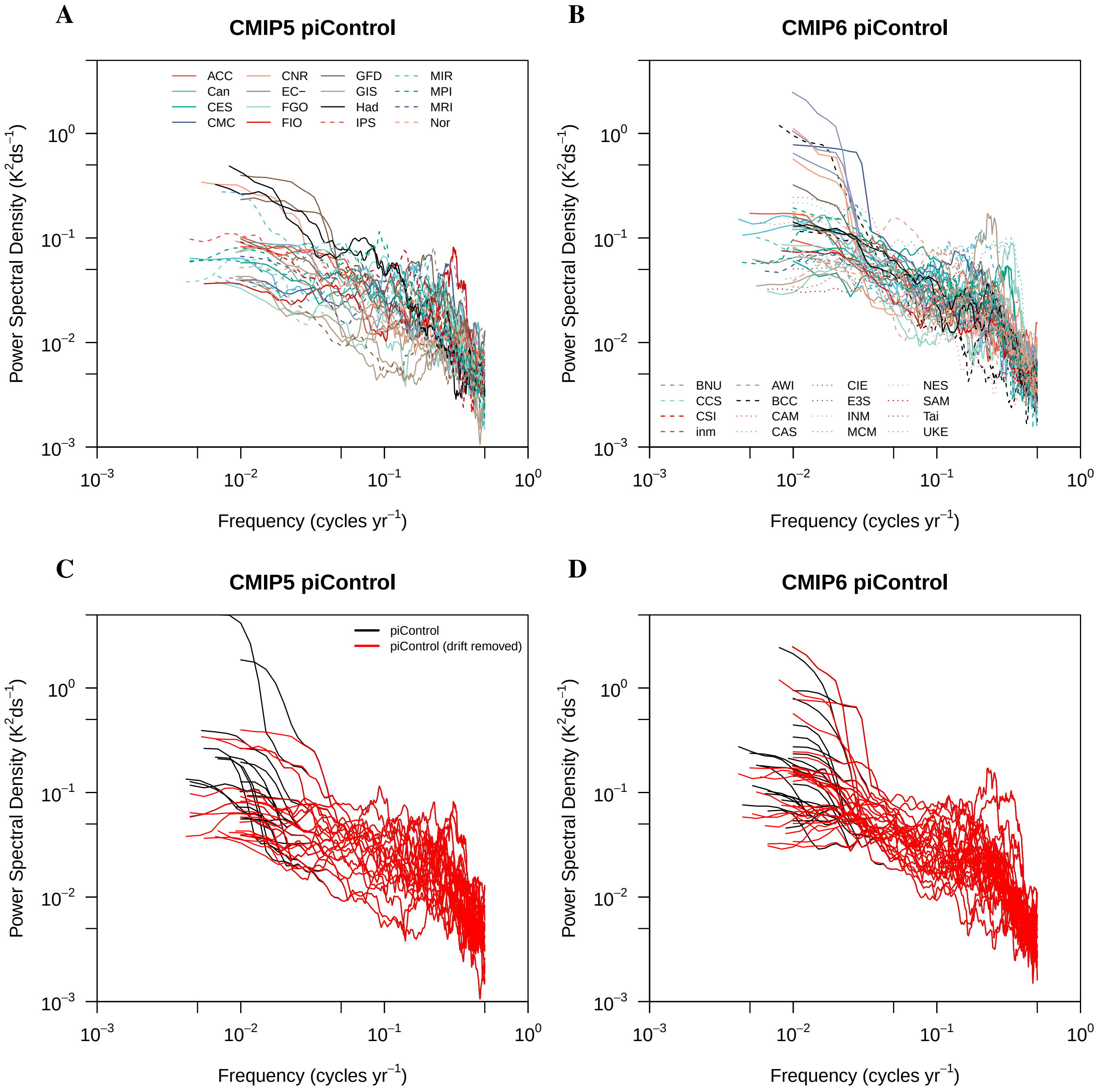

Fig. S1. Power spectra of climate model pre-industrial control simulations with at least $N = 350$ simulation years from the (A, C) CMIP5 and (B, D) CMIP6 archives. (A, B) Power spectra of individual climate models' pre-industrial control simulations (drift removed), and (C, D) climate model pre-industrial control simulations with and without drift removal. Power spectra are computed from a raw periodogram that is subsequently smoothed using a Daniell kernel of width $M = 15$, using the R function 'spec.pgram'. Spectral density estimates for the three lowest frequencies are not shown to avoid overinterpretation.

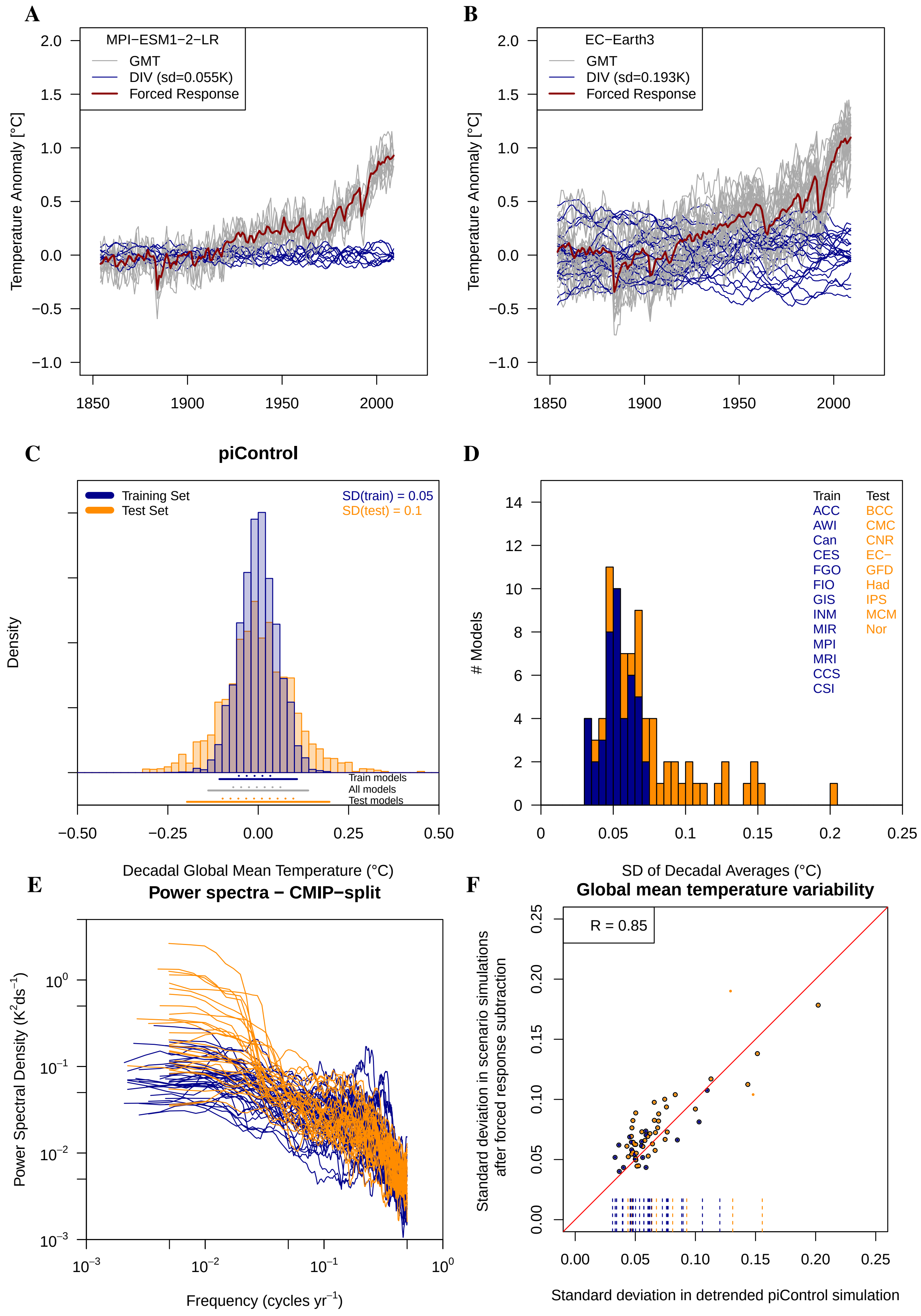
A
MPI−ESM1−2−LR
GMT
DIV (sd=0.055K)
Forced Response
Temperature Anomaly [°C]
2.0
1.5
1.0
0.5
0.0
−0.5
−1.0
1850
1900
1950
2000
B
EC−Earth3
GMT
DIV (sd=0.193K)
Forced Response
Temperature Anomaly [°C]
C
piControl
Training Set
Test Set
SD(train) = 0.05
SD(test) = 0.1
Density
Train models
All models
Test models
−0.50
−0.25
0.00
0.25
0.50
Decadal Global Mean Temperature (°C)
D
Train
ACC
AWI
Can
CES
FGO
FIO
GIS
INM
MIR
MPI
MRI
CCS
CSI
Test
BCC
CMC
CNR
EC−
GFD
Had
IPS
MCM
Nor
Models
14
12
10
8
6
4
2
0
0
0.05
0.1
0.15
0.2
0.25
SD of Decadal Averages (°C)
E
Power spectra − CMIP−split
Power Spectral Density (K^{2}ds^{-1})
10^0
10^{-1}
10^{-2}
10^{-3}
Frequency (cycles yr^{-1})
F
Global mean temperature variability
R = 0.85
Standard deviation in scenario simulations after forced response subtraction
0.00
0.05
0.10
0.15
0.20
0.25
Standard deviation in detrended piControl simulation

Fig. S2. Illustration of the 'CMIP train-test split' experiment and separation of forced response and internal variability. (A, B) For each climate model, we estimate the externally forced global mean temperature response by averaging across all available ensemble members for both historical and scenario simulations (see Material&Methods for details; illustrated here only for historical simulations up to 2014). We obtain an estimate of global decadal-scale internal variability (DIV) as a 10-year moving average of the difference between each ensemble member's GMT and the forced response. The separation between the forced response and DIV is illustrated for a model with relatively modest DIV (A, MPI-ESM1-2-LR), and a model that shows high DIV (B, EC-Earth3). (C) Histograms of unforced decadal temperature anomalies for low-variability training models (blue) and high-variability test models (orange). (D) For each model that we analyzed in the CMIP5 and CMIP6 archive, we calculate the standard deviation of DIV in the preindustrial control simulations (where any forced response is absent). We split models into a set of test models with high decadal-scale internal variability (orange, including *all* other model variants) and a set of low-variability training models (blue). Differences in the shading of blue and orange colors in (C) and (D) are to simplify visual interpretation, as (C) shows two separate histograms that are overlaid, while (D) shows one stacked histogram made up of training and test models. (E) Power spectra for each model variant's preindustrial control simulations (similar to Fig. S1), but split by low-variability models (shown in blue) and high-variability models (shown in orange). (F) Scatterplot across all CMIP5 and CMIP6 model variants between DIV estimates extracted from preindustrial control simulations (x-axis) and DIV estimated from historical and scenario simulations where the multi-member mean (a proxy of the forced response) is subtracted (y-axis).

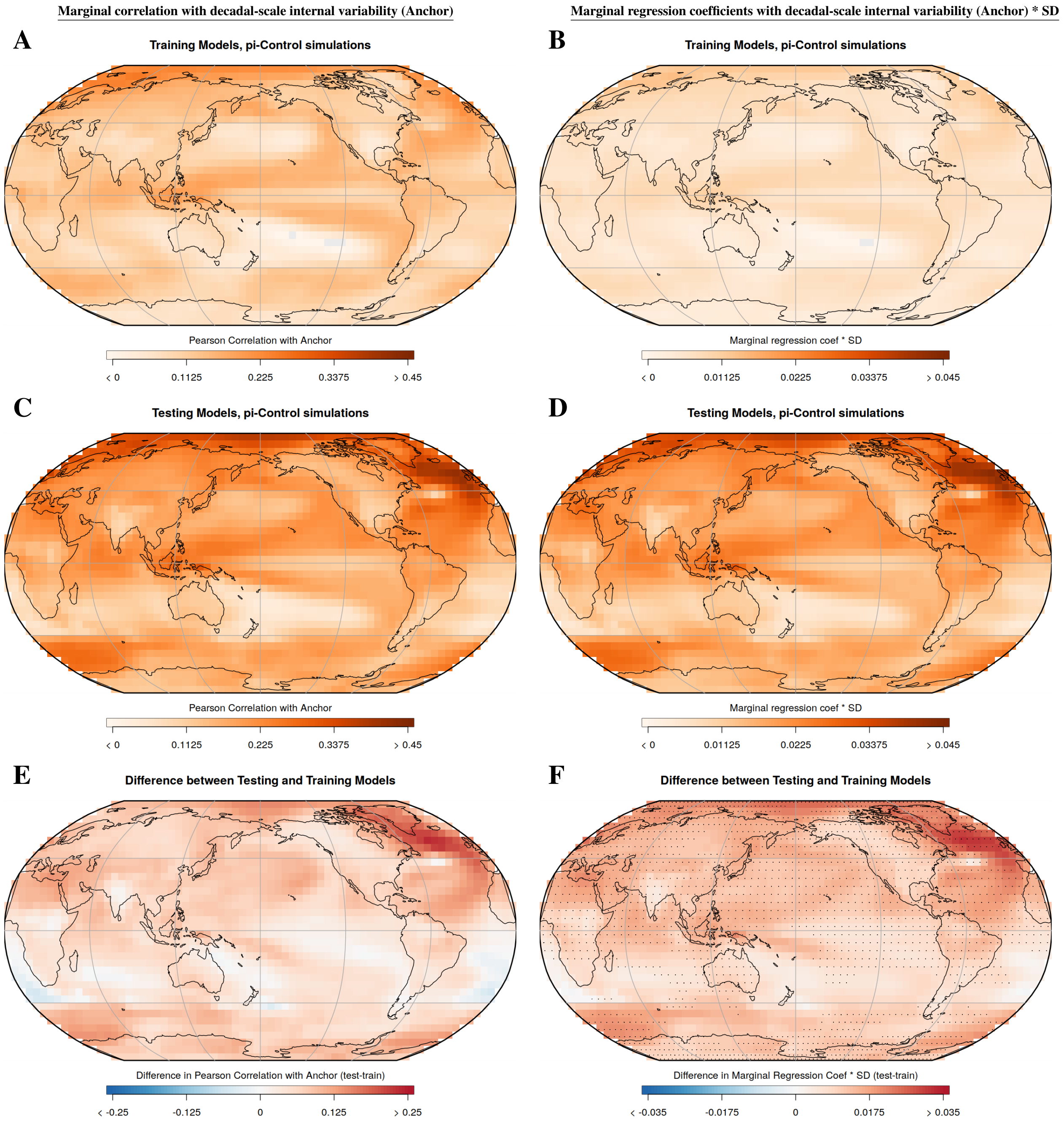

Fig. S3. Comparison of modes of internal variability in preindustrial control simulations of low-variability models and high-variability models. (A, C, E) Marginal (grid point based) correlation between annual GMT and global decadal-scale internal variability (DIV, i.e., the anchor variable), averaged across (A) low-variability, and (C) high-variability models, and (E) the difference between high-variability and low-variability models. (B, D, F) Marginal (grid point based) linear regression coefficients obtained from regressing DIV on local annual GMT, multiplied by the local standard deviation of annual GMT, for (B) low-variability, and (D) high-variability models, and (F) the difference between high-variability and low-variability models. Stippling in the bottom panels indicates locally significant differences between the averages of high-variability and low-variability models, based on a non-parametric Wilcoxon rank-sum test, with control of the false discovery rate at the $\alpha_0 = 5\%$ level as described in Wilks (2016), *Bulletin of the American Meteorological Society* **97**, 2263, doi:10.1175/BAMS-D-15-00267.1.

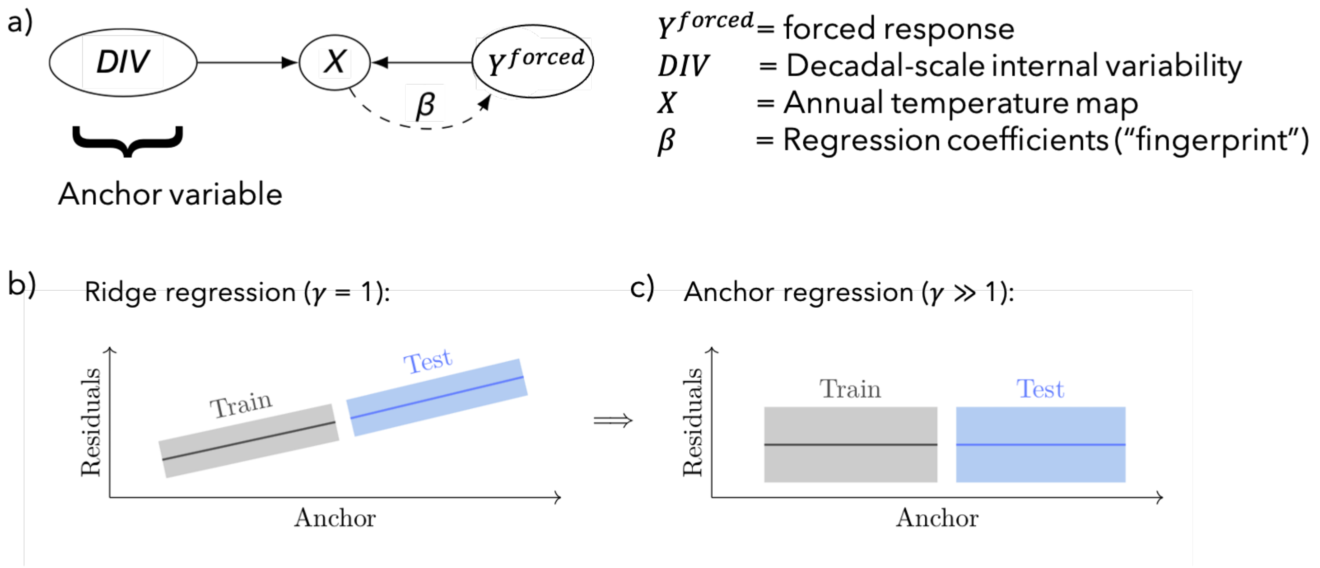

Fig. S4. Illustration of detection and attribution methodology and anchor regression. (a) The goal of our detection and attribution method is to predict a forced response proxy Y^{forced} from a global annual temperature map X using a set of regression coefficients β ('fingerprint', see Materials and Methods for detailed description). Decadal-scale internal variability (DIV), e.g., via decadal or multidecadal modes of internal climate variability, affects annual temperatures, and is used as the anchor variable in the anchor regression method used for fingerprint extraction. (b) In linear regression without a specific anchor variable (e.g., in ridge regression with $\gamma = 1$), variation in the anchor variable may induce a correlation between the anchor variable and the residuals of the prediction; in particular if the anchor variable would be larger in a testing setting than in a training setting. For example, in the climate context illustrated in this paper, large DIV in test models may project onto the fingerprint of the forced response and thereby distort detection results. (c) The rationale for our use of anchor regression is to employ variation in the anchor variable in the training context in order to reduce the correlation between the anchor variable and prediction residuals, thereby increasing the robustness of the fingerprints of the forced response to large variability in the anchor variable. These basic features of anchor regression are further illustrated in Fig. 2 in the main manuscript and in Fig. S6, and are explained in detail in the Material and Methods section.

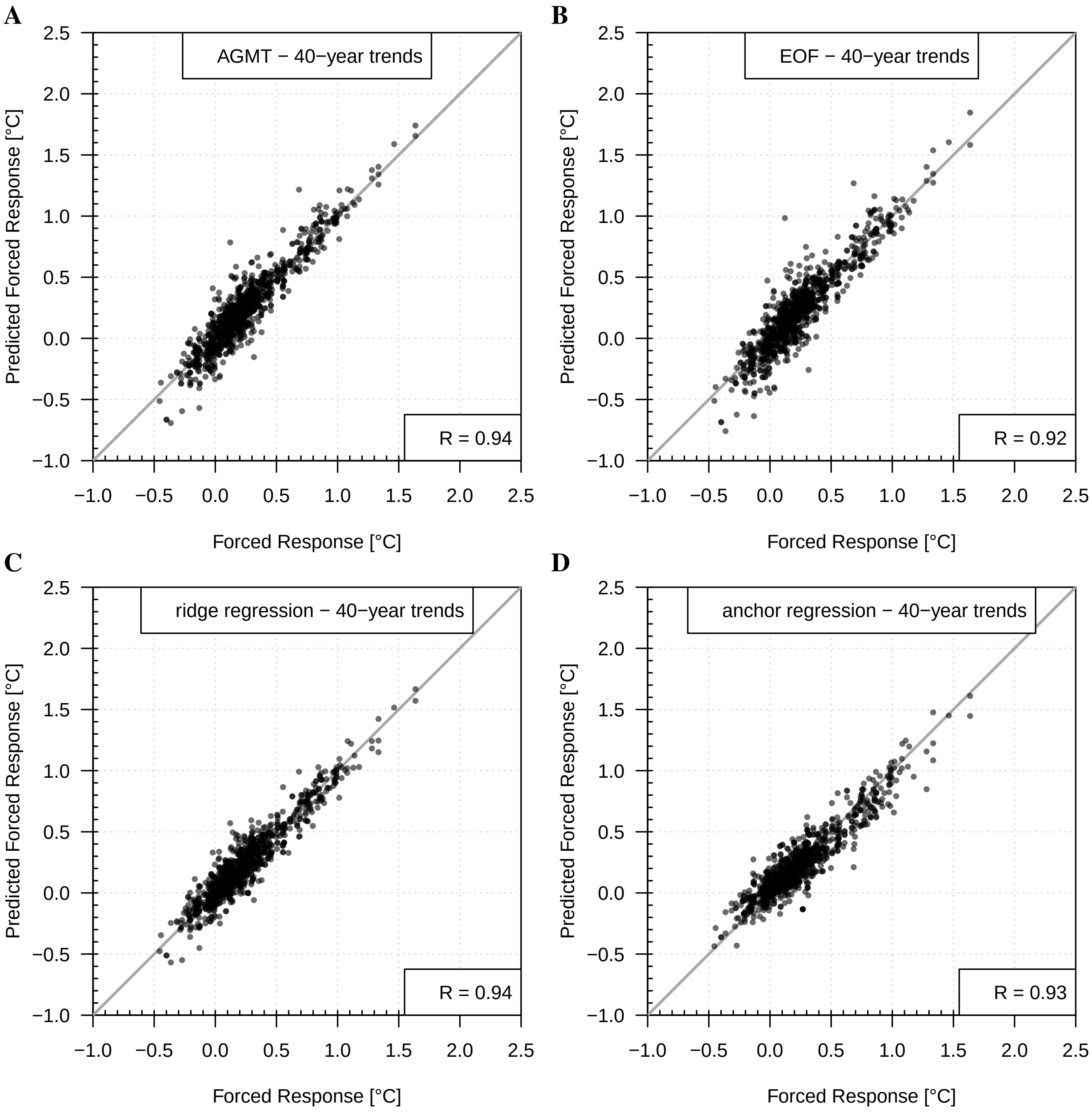

Fig. S5. Forced 40-year trends (extracted from each climate model) vs. reconstruction (prediction) of forced 40-year trends in historical and scenario simulations using predictions made by (A) GMT detection metric, (B) the mean warming pattern (MWP detection metric), (C) ridge regression, and (D) anchor regression (Anchor 0.5/0.5).

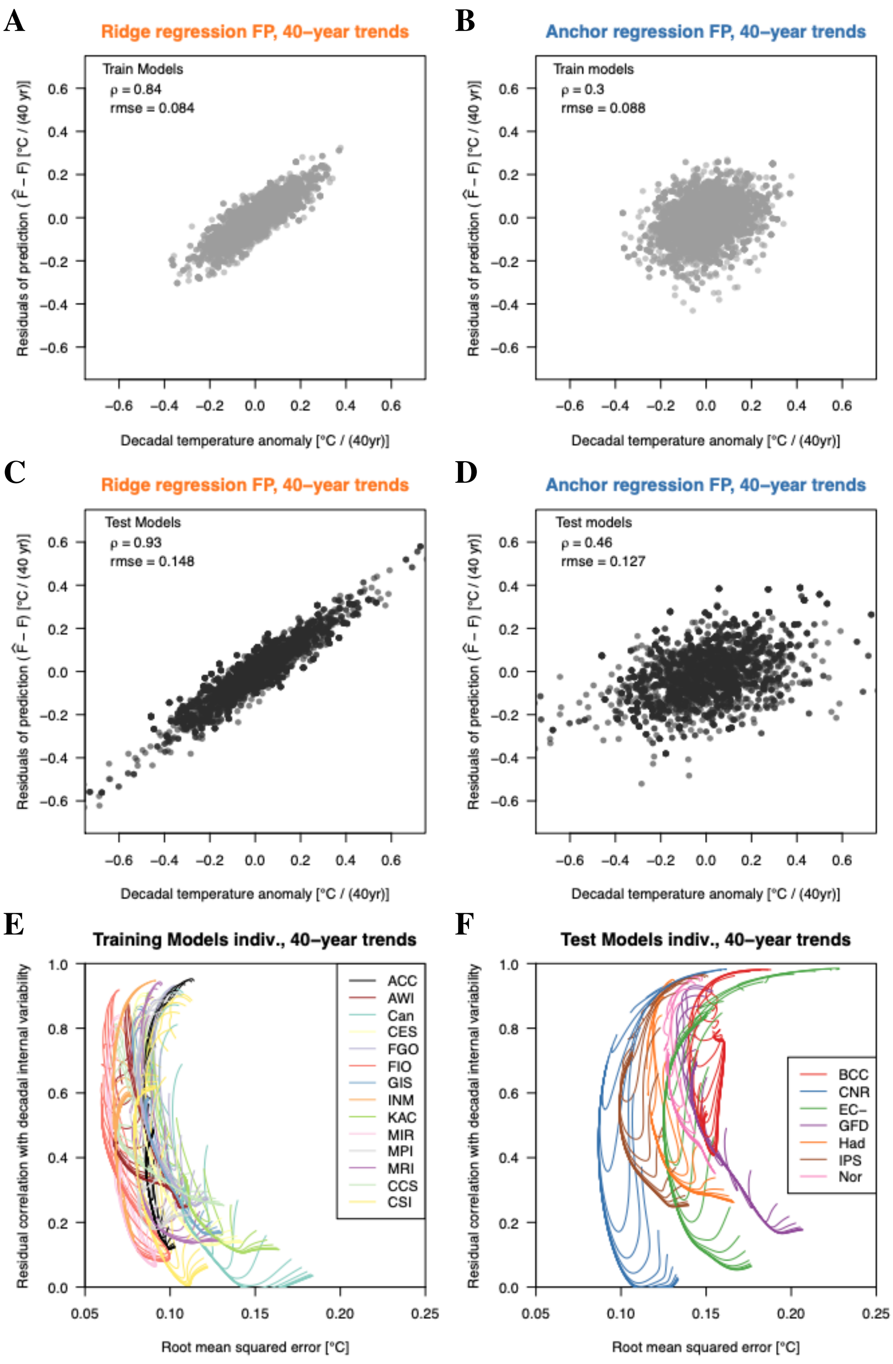

Fig. S6. Illustration of correlation of unforced 40-year trends with prediction residuals ('residual correlation'). Residual correlation shown for (A,B) training models and (C, D) test models for both (A, C) ridge regression predictions and (B, D) anchor regression predictions. (E, F) RMSE in estimating 40-year forced temperature trends and the correlation of prediction residuals ($\hat{Y}^{\text{forced}} - Y^{\text{forced}}$ with DIV for (E) each training model and (F) each test model in the 'cmip train-test split' experiment.

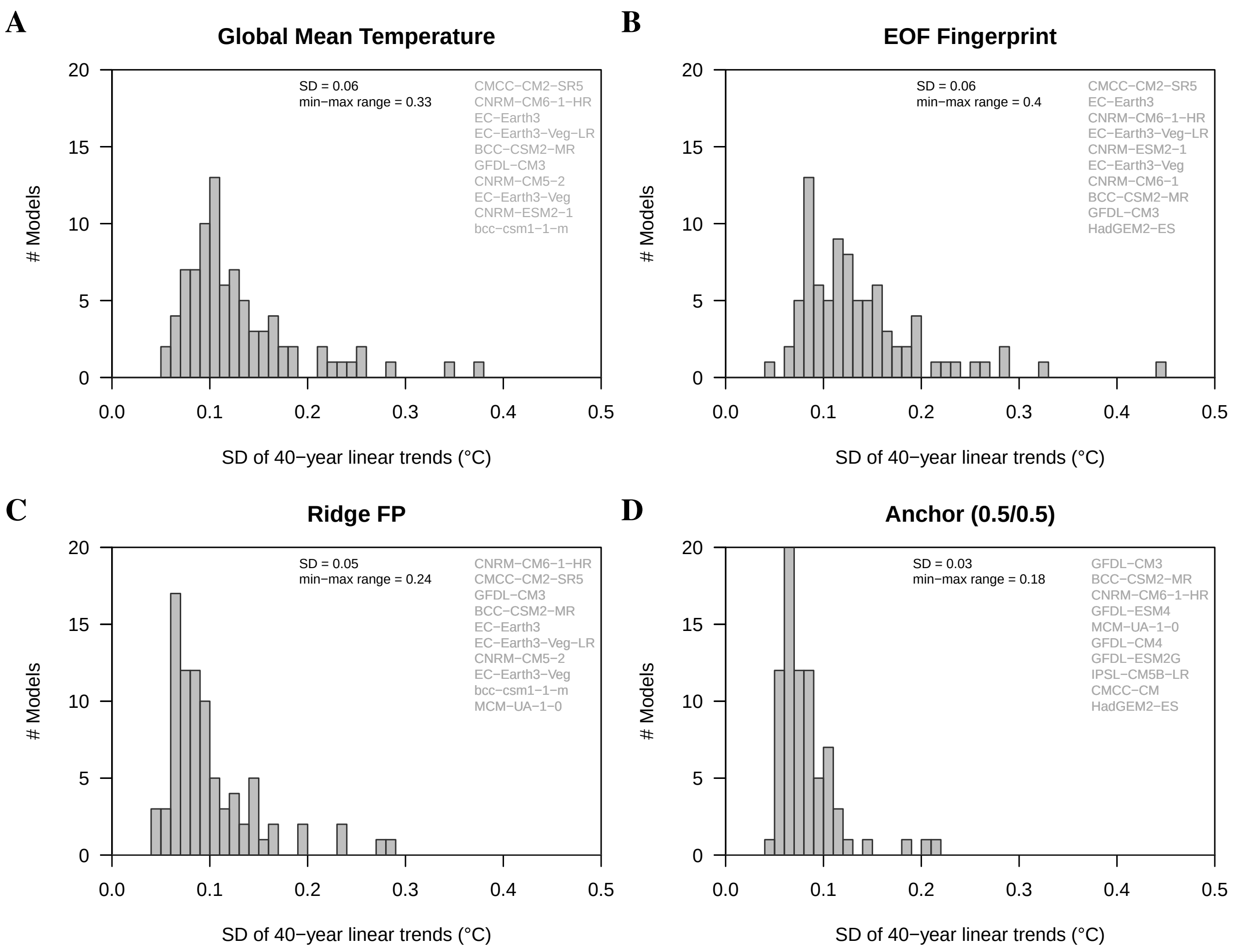

Fig. S7. Histograms of standard deviation of 40-year linear trends in pre-industrial control simulations for each climate model, and for different detection metrics: (A) Global mean temperature, (B) mean warming pattern, (C) ridge regression, and (D) the anchor regression detection metric.

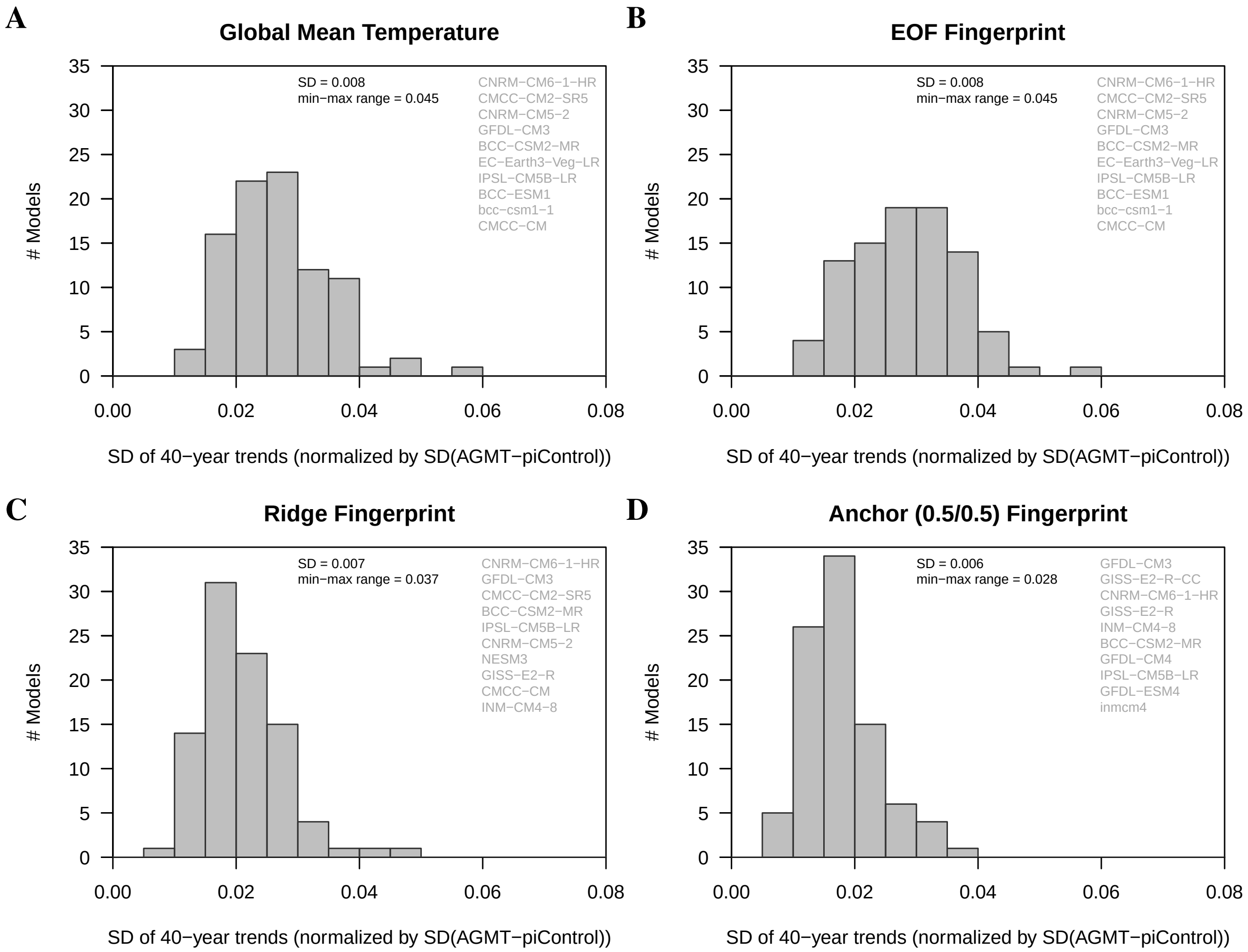

Fig. S8. Histograms of standard deviation of 40-year linear trends in pre-industrial control simulations for each climate model, normalized to the same annual global mean temperature SD, and for different detection metrics: (A) Global mean temperature, (B) mean warming pattern, (C) ridge regression, and (D) the anchor regression detection metric.

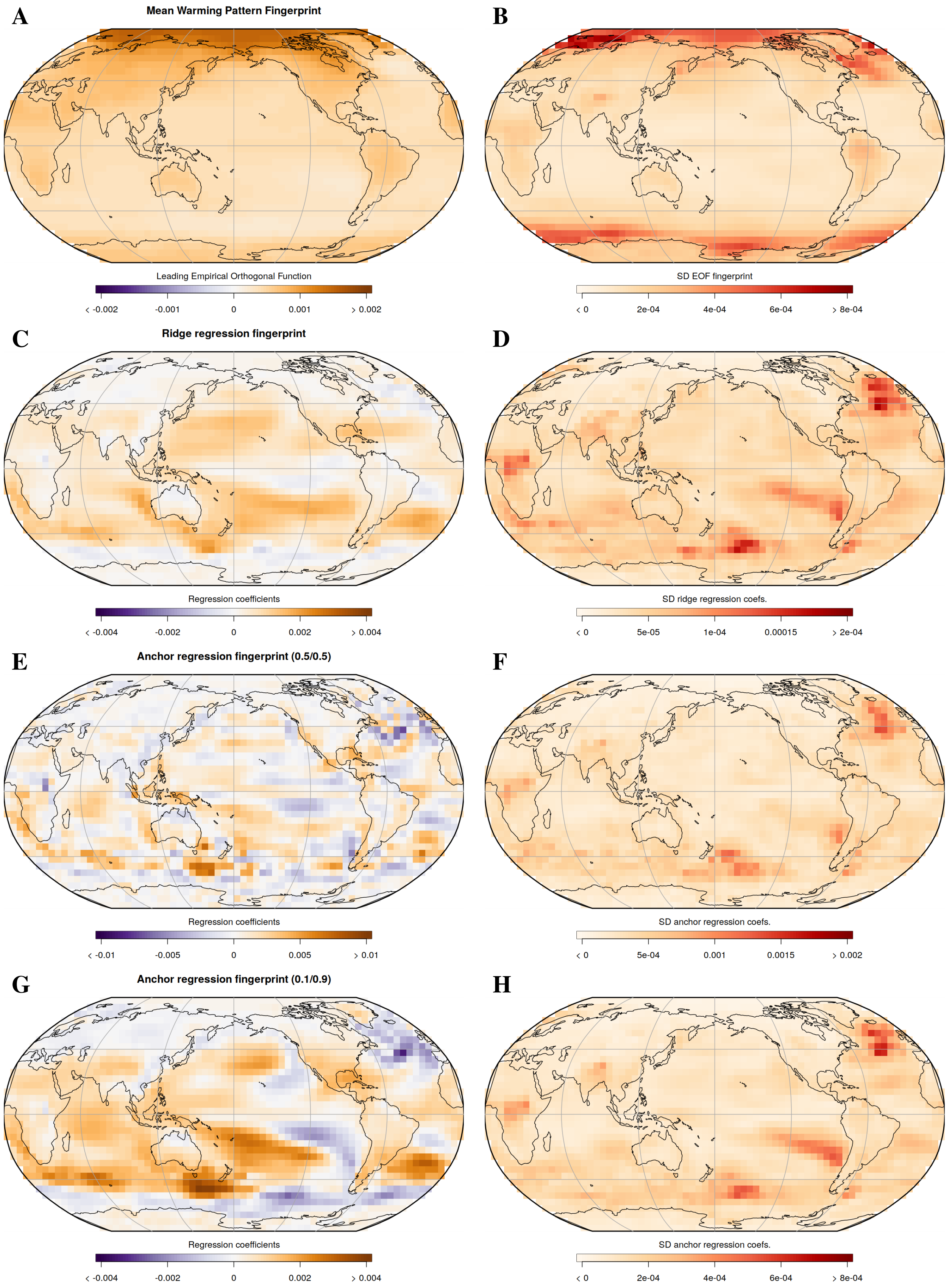

Fig. S9. Illustration of (A,C,E,G) fingerprints and (B,D,F,H) associated uncertainty (standard deviation across different individual fingerprints from fingerprint extraction, see Materials&Methods for details) in each grid cell. Fingerprints include the mean warming pattern (A, B), ridge regression (C, D, $\lambda = 10^{4.36}$), anchor regression (0.5/0.5) (E, F, $\lambda = 10^{3.45}$, $\gamma = 50$), and anchor regression (0.1/0.9) (G, H, $\lambda = 10^{4.61}$, $\gamma = 500$).

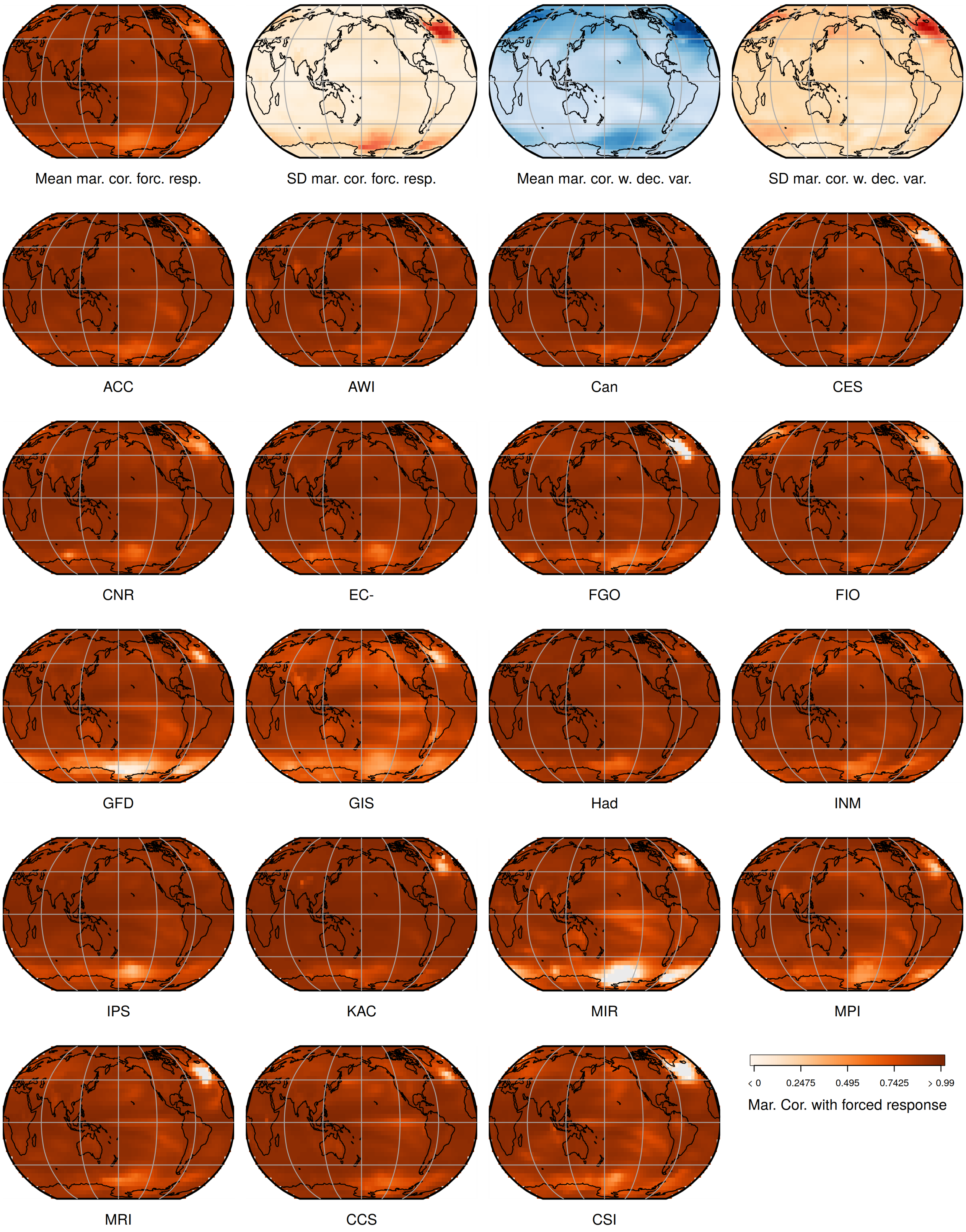

Fig. S10. (top row, from left to right) Correlation of local temperatures with the forced response across CMIP models, standard deviation of correlation after leave-one-out evaluation, correlation of local temperatures with decadal-scale internal variability (DIV) across pre-industrial control simulations of CMIP models, and standard deviation of correlation after leave-one-out evaluation. (second to last row) Correlation of local temperatures with the forced response across individual models.

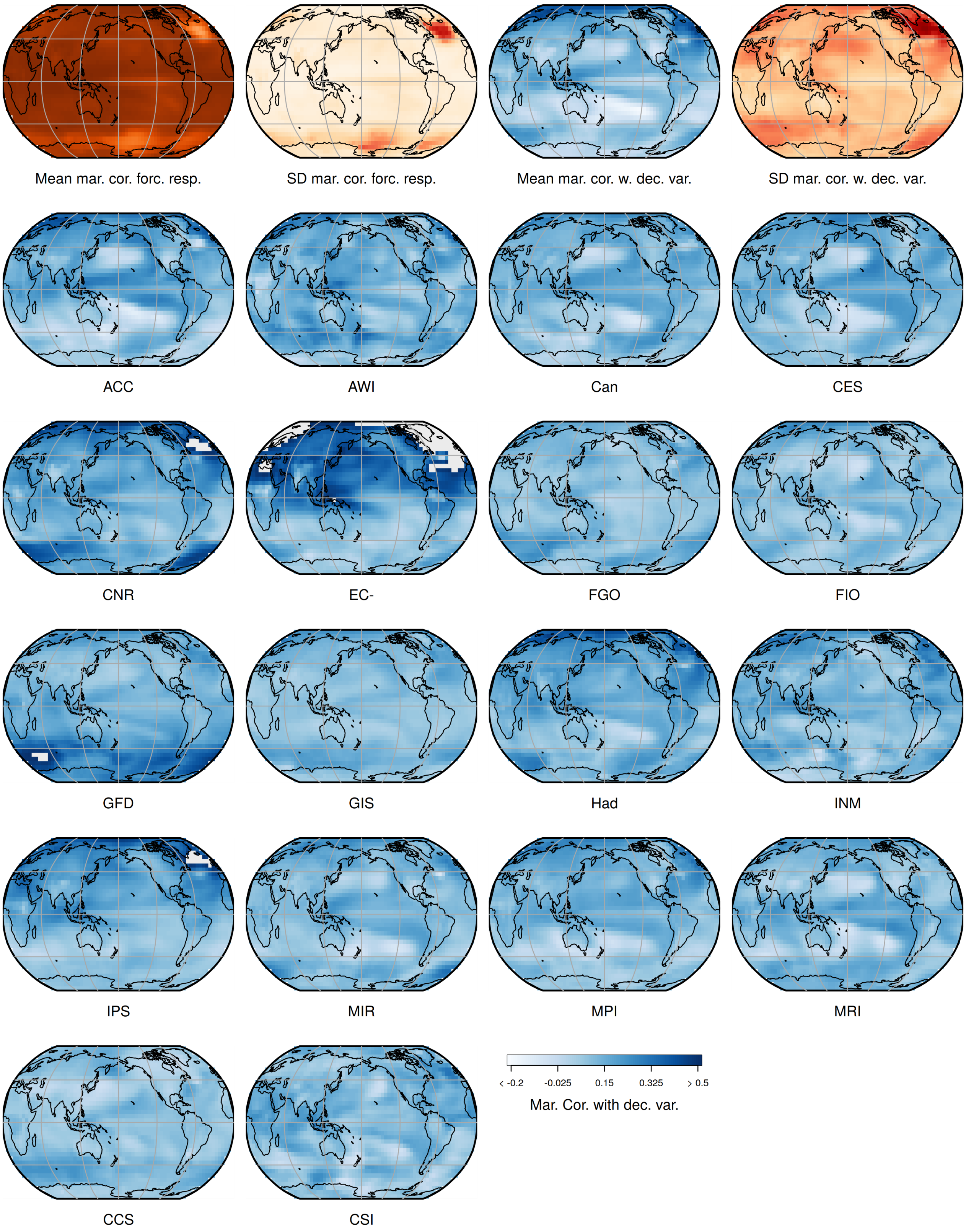

Fig. S11. (top row, from left to right) Correlation of local temperatures with the forced response across CMIP models, standard deviation of correlation after leave-one-out evaluation, correlation of local temperatures with decadal-scale internal variability (DIV) across pre-industrial control simulations of CMIP models, and standard deviation of correlation after leave-one-out evaluation. (second to last row) Correlation of local temperatures with decadal-scale internal variability (DIV) across pre-industrial control simulations of individual CMIP models.

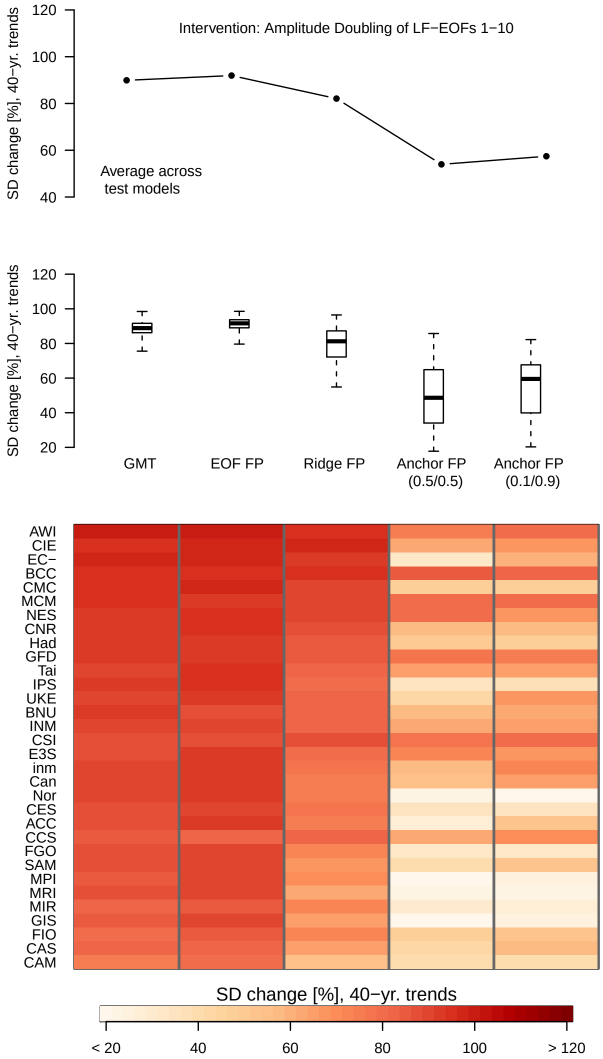

Fig. S12. Relative change (in %) in the standard deviation of 40-year trends in pre-industrial control simulations upon distributional changes of the main modes of DIV, shown for each model individually and five different D&A fingerprints. After doubling of the magnitude of the first 10 Empirical Orthogonal Functions ('scaled DIV'), the standard deviation of 40-year pre-industrial control trends increases, but the increase is more modest for the anchor regression fingerprints.

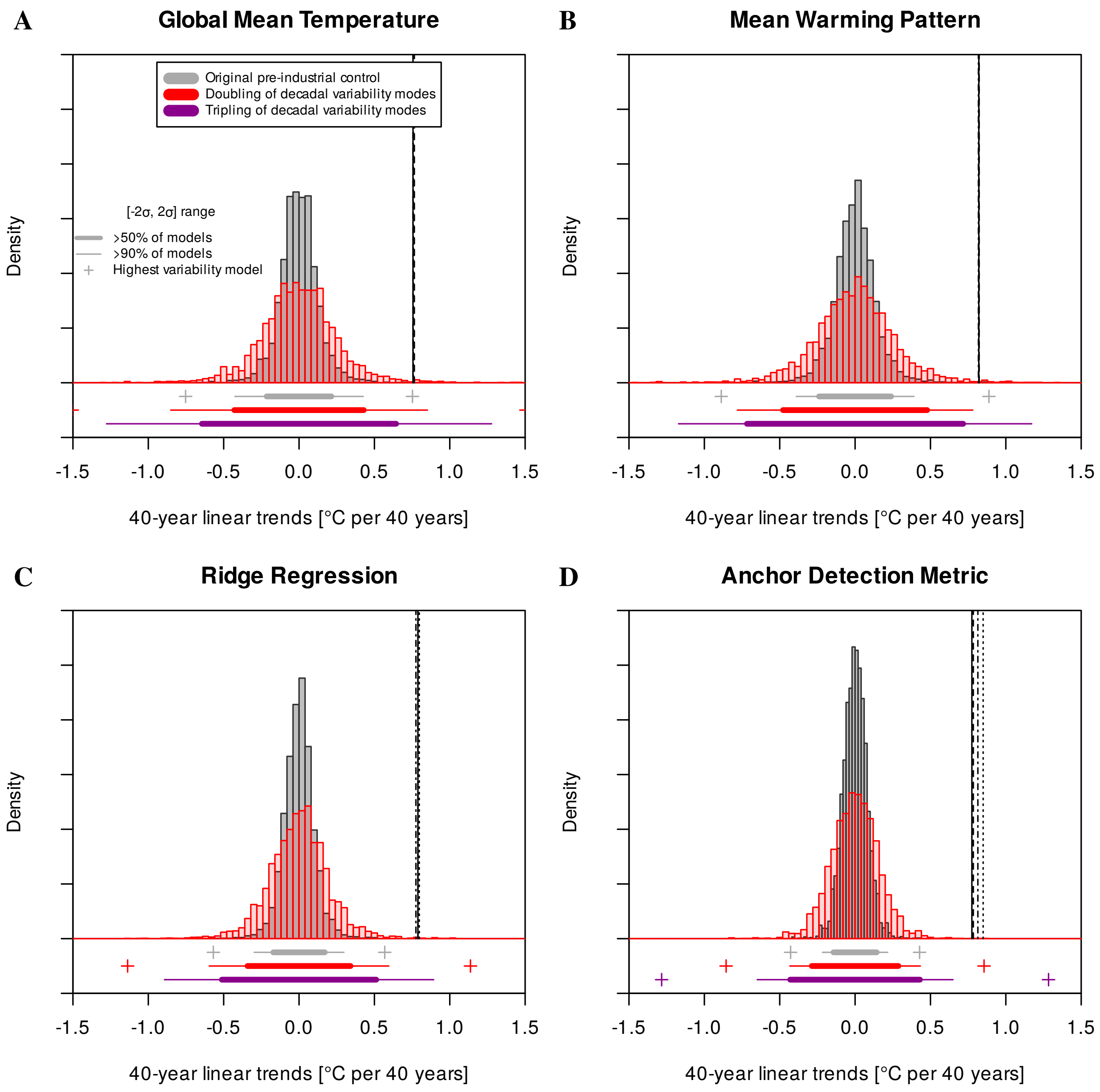

Fig. S13. Detection of observed 40-year temperature trend (1980–2019) in the presence of potentially large decadal-scale internal variability under doubling and tripling of the standard deviation of internal variability. Histogram of 40-year trends from preindustrial control simulations for the (A) global mean temperature, (B) mean warming pattern, (c) ridge regression, and (D) anchor regression (0.5/0.5) detection metrics, shown alongside observed and reanalyzed 1980-2019 trends (black vertical lines). This figure differs to Fig. 5 in the main text in that we show here results for a doubling and tripling of the standard deviation, instead of a doubling or tripling only of the standard deviation of the first 10 EOF modes (which is shown in Fig. 5 in the main text).

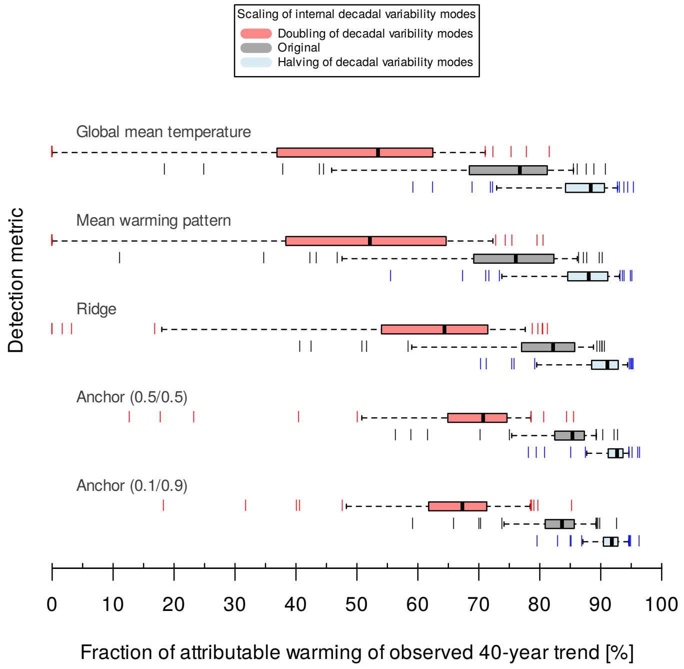

Fig. S14. As Fig. 6B in the main paper, but for halving and doubling the standard deviation of internal variability.

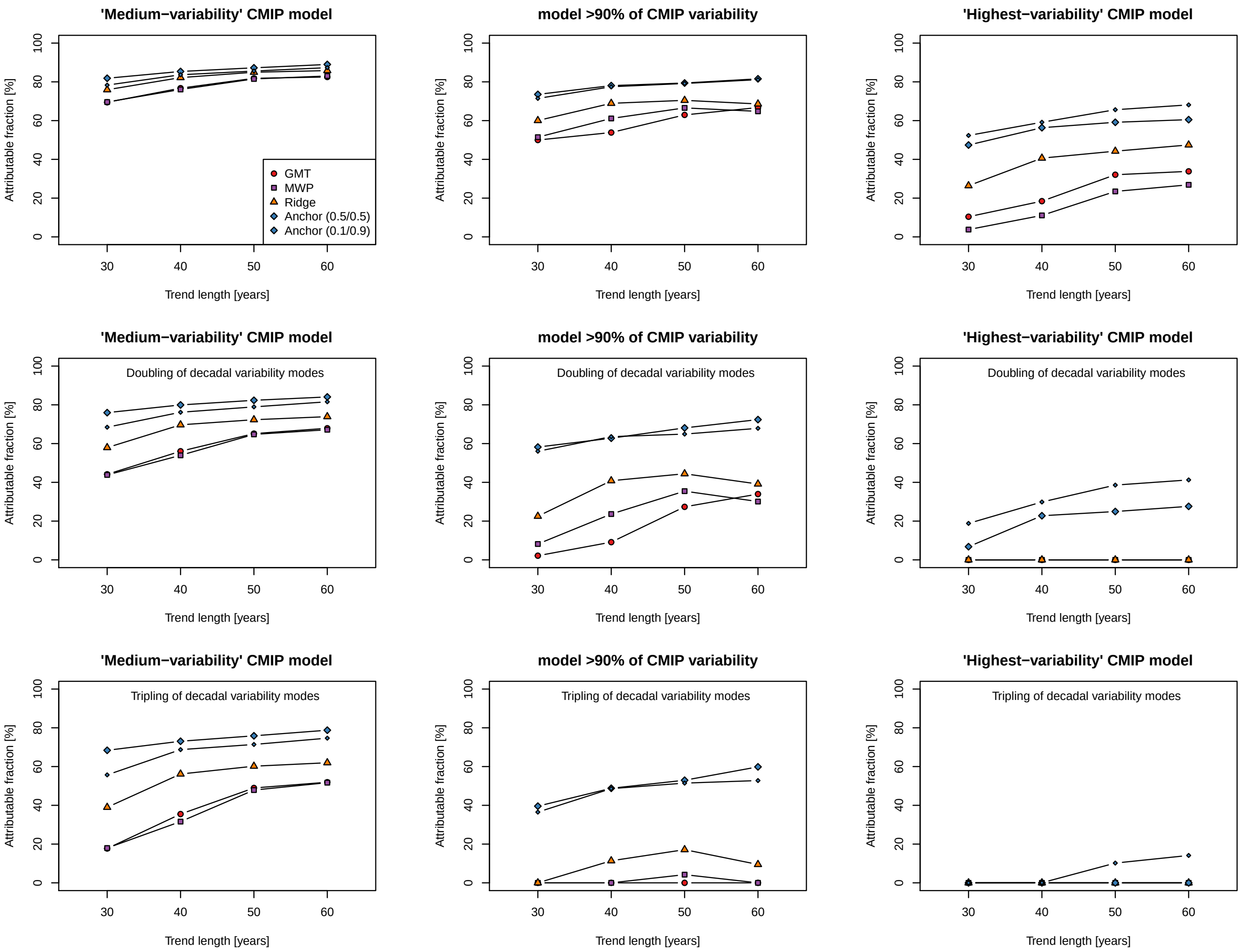

Fig. S15. Attributable warming fraction as a function of the trend time scale. Shown for (left panels) median-variability CMIP model, (middle panels) high-variability ($> 90\%$) CMIP model, and (right panels) highest-variability CMIP model; and for (top) original variability of pre-industrial control simulations, (middle) doubling of the main modes of decadal variability, and (bottom) tripling of the main modes of decadal variability. All plots show attributable warming fractions based on the *'extremely likely'* level.

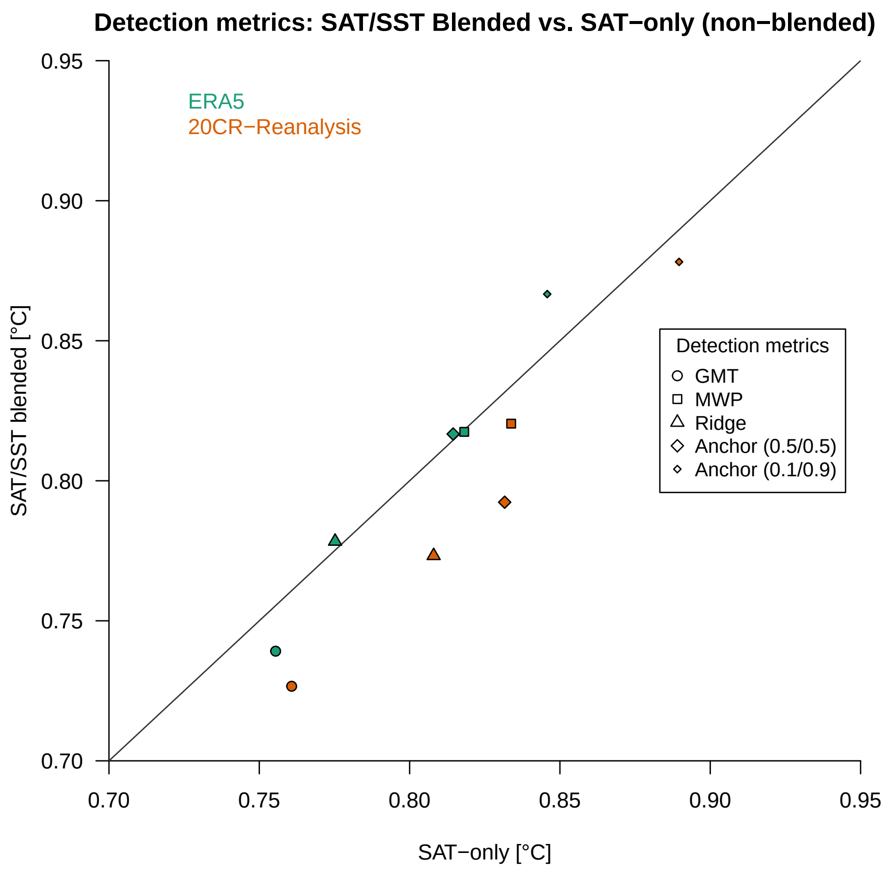

Fig. S16. Comparison of detection metrics based on non-blended (surface air temperature, SAT) and blended (SAT over land, skin temperature over ocean) reanalysis data. Differences are very small in ERA5, and slightly larger in the Twentieth Century Reanalysis (Version3). Detection based on blended data is conservative.

Supplementary Tables

Table 1: Overview of CMIP5 and CMIP6 models used in the analysis.

Model	Model family	CMIP	Scenario & Number of ensembe members	train & test split
ACCESS-CM2	ACC	cmip6	historical(3), SSP126(3), SSP245(3), SSP370(3), SSP585(3)	train
ACCESS-ESM1-5	ACC	cmip6	historical(10), SSP126(3), SSP245(3), SSP370(3), SSP585(3)	train
AWI-CM-1-1-MR	AWI	cmip6	historical(5), SSP370(5)	train
BCC-CSM2-MR	BCC	cmip6	historical(3)	test
BCC-ESM1	BCC	cmip6	historical(3)	test
CAMS-CSM1-0	CAM	cmip6	historical(3)	-
CanESM5	Can	cmip6	historical(65), SSP119(50), SSP126(50), SSP245(50), SSP370(50), SSP434(5), SSP585(50)	train
CanESM5-CanOE	Can	cmip6	historical(3), SSP126(3), SSP245(3), SSP370(3), SSP585(3)	train
CanESM2	Can	cmip5	historical(5), RCP26(5), RCP45(5), RCP85(5)	train
CAS-ESM2-0	CAS	cmip6	historical(4)	-
CESM2	CES	cmip6	historical(11), SSP126(5), SSP245(6), SSP370(8), SSP585(5)	train
CESM2-FV2	CES	cmip6	historical(3)	train
CESM2-WACCM	CES	cmip6	historical(3), SSP245(5), SSP585(5)	train
CESM2-WACCM-FV2	CES	cmip6	historical(3)	train
CESM1-CAM5	CES	cmip5	historical(3), RCP26(3), RCP45(3), RCP85(3)	train
CIESM	CIE	cmip6	historical(3)	-
CNRM-CM6-1	CNR	cmip6	historical(30), SSP126(6), SSP245(6), SSP370(6), SSP585(6)	test
CNRM-ESM2-1	CNR	cmip6	historical(10), SSP119(5), SSP126(5), SSP245(5), SSP370(5), SSP434(5), SSP585(5)	test
CNRM-CM5	CNR	cmip5	historical(5), RCP85(5)	test
E3SM-1-0	E3S	cmip6	historical(5)	-
EC-Earth3	EC-	cmip6	historical(23), SSP119(51), SSP126(7), SSP245(22), SSP370(7), SSP434(50), SSP585(57)	test
EC-Earth3-Veg	EC-	cmip6	historical(6), SSP119(3), SSP126(5), SSP245(5), SSP370(4), SSP585(5)	test
EC-Earth3-Veg-LR	EC-	cmip6	historical(3)	test
EC-EARTH	EC-	cmip5	historical(6), RCP45(6), RCP85(6)	test
FGOALS-f3-L	FGO	cmip6	historical(3)	train
FGOALS-g3	FGO	cmip6	historical(6), SSP245(4), SSP370(5), SSP585(4)	train
FIO-ESM-2-0	FIO	cmip6	historical(3), SSP126(3), SSP245(3), SSP585(3)	train
FIO-ESM	FIO	cmip5	historical(3), RCP26(3), RCP45(3), RCP85(3)	train
GFDL-ESM4	GFD	cmip6	historical(3), SSP245(3)	test
GFDL-CM3	GFD	cmip5	historical(3), RCP45(3)	test
GISS-E2-1-G	GIS	cmip6	historical(40), SSP245(5), SSP370(7)	train
GISS-E2-1-H	GIS	cmip6	historical(23)	train
GISS-E2-H	GIS	cmip5	historical(15), RCP26(3), RCP45(15), RCP85(5)	train
GISS-E2-R	GIS	cmip5	historical(17), RCP26(3), RCP45(17), RCP85(5)	train
HadGEM3-GC31-LL	Had	cmip6	historical(4), SSP585(4)	test
HadGEM3-GC31-MM	Had	cmip6	historical(4), SSP585(3)	test
UKESM1-0-LL	Had	cmip6	historical(18), SSP119(5), SSP126(13), SSP245(5), SSP370(13), SSP434(5), SSP585(5)	test
HadGEM2-ES	Had	cmip5	historical(4), RCP26(4), RCP45(4), RCP85(4)	test
INM-CM5-0	INM	cmip6	historical(10), SSP370(5)	train
IPSL-CM6A-LR	IPS	cmip6	historical(32), SSP119(6), SSP126(6), SSP245(11), SSP370(11), SSP585(6)	test
IPSL-CM5A-LR	IPS	cmip5	historical(4), RCP26(4), RCP45(4), RCP85(4)	test
KACE-1-0-G	KAC	cmip6	historical(3), SSP126(3), SSP245(3), SSP370(3), SSP585(3)	train

MIROC-ES2L	MIR	cmip6	historical(10), SSP119(3), SSP126(3)	train
MIROC6	MIR	cmip6	historical(50), SSP126(11), SSP245(3), SSP370(3), SSP585(50)	train
MIROC5	MIR	cmip5	historical(3), RCP26(3), RCP45(3), RCP85(3)	train
MPI-ESM1-2-HR	MPI	cmip6	historical(10), SSP370(10)	train
MPI-ESM1-2-LR	MPI	cmip6	historical(10), SSP126(10), SSP245(10), SSP370(10), SSP585(10)	train
MPI-ESM-LR	MPI	cmip5	historical(3), RCP26(3), RCP45(3), RCP85(3)	train
MPI-ESM-MR	MPI	cmip5	historical(3), RCP45(3)	train
MRI-ESM2-0	MRI	cmip6	historical(6), SSP370(5)	train
NESM3	NES	cmip6	historical(5)	-
NorESM2-LM	Nor	cmip6	historical(3), SSP245(3)	test
CCSM4	CCS	cmip5	historical(6), RCP26(6), RCP45(6), RCP85(6)	train
CSIRO-Mk3-6-0	CSI	cmip5	historical(10), RCP26(10), RCP45(10), RCP85(10)	train
CSIRO-Mk3L-1-2	CSI	cmip5	historical(3), RCP45(3)	train

Table 2: Internal variability estimates (standard deviation of 40-year trends) from CMIP5 and CMIP6 pre-industrial control simulations

Model	Model family[a]	CMIP	train& test split	sd(AGMT)	sd(AGMT-DIV doubl.)	sd(anchor0.5)	sd(anchor0.5-DIV doubl.)
ACCESS-CM2	ACC	cmip6	train	0.11	0.2	0.07	0.08
ACCESS-ESM1-5	ACC	cmip6	train	0.12	0.23	0.06	0.07
ACCESS1-0	ACC	cmip5	train	0.11	0.2	0.07	0.08
ACCESS1-3	ACC	cmip5	train	0.11	0.21	0.06	0.08
AWI-CM-1-1-MR	AWI	cmip6	train	0.12	0.24	0.09	0.16
BCC-CSM2-MR	BCC	cmip6	test	0.26	0.51	0.2	0.37
BCC-ESM1	BCC	cmip6	test	0.14	0.26	0.06	0.11
bcc-csm1-1	BCC	cmip5	test	0.15	0.27	0.05	0.1
bcc-csm1-1-m	BCC	cmip5	test	0.21	0.42	0.11	0.21
CAMS-CSM1-0	CAM	cmip6	-	0.08	0.14	0.06	0.09
CanESM5	Can	cmip6	train	0.12	0.23	0.1	0.15
CanESM5-CanOE	Can	cmip6	train	0.13	0.25	0.08	0.14
CanESM2	Can	cmip5	train	0.08	0.14	0.07	0.09
CAS-ESM2-0	CAS	cmip6	-	0.1	0.18	0.06	0.08
CESM2	CES	cmip6	train	0.12	0.22	0.08	0.11
CESM2-FV2	CES	cmip6	train	0.09	0.16	0.07	0.1
CESM2-WACCM	CES	cmip6	train	0.12	0.23	0.07	0.09
CESM2-WACCM-FV2	CES	cmip6	train	0.11	0.21	0.05	0.07
CESM1-BGC	CES	cmip5	train	0.08	0.16	0.07	0.1
CESM1-CAM5	CES	cmip5	train	0.11	0.2	0.06	0.09
CESM1-FASTCHEM	CES	cmip5	train	0.1	0.2	0.04	0.06
CIESM	CIE	cmip6	-	0.09	0.18	0.06	0.09
CMCC-CM2-SR5	CMC	cmip6	test	0.38	0.74	0.09	0.13
CMCC-CESM	CMC	cmip5	test	0.1	0.19	0.08	0.11
CMCC-CM	CMC	cmip5	test	0.16	0.31	0.11	0.16
CMCC-CMS	CMC	cmip5	test	0.07	0.13	0.06	0.09
CNRM-CM6-1	CNR	cmip6	test	0.18	0.36	0.08	0.12
CNRM-CM6-1-HR	CNR	cmip6	test	0.35	0.66	0.19	0.28
CNRM-ESM2-1	CNR	cmip6	test	0.21	0.43	0.08	0.12
CNRM-CM5	CNR	cmip5	test	0.16	0.31	0.08	0.14
CNRM-CM5-2	CNR	cmip5	test	0.24	0.45	0.09	0.13
E3SM-1-0	E3S	cmip6	-	0.11	0.2	0.09	0.16
EC-Earth3	EC-	cmip6	test	0.29	0.57	0.07	0.09
EC-Earth3-Veg	EC-	cmip6	test	0.22	0.44	0.05	0.07
EC-Earth3-Veg-LR	EC-	cmip6	test	0.26	0.51	0.07	0.09
EC-EARTH	EC-	cmip5	test	0.12	0.22	0.07	0.1
FGOALS-f3-L	FGO	cmip6	train	0.11	0.2	0.08	0.09
FGOALS-g3	FGO	cmip6	train	0.08	0.15	0.05	0.06
FGOALS-g2	FGO	cmip5	train	0.06	0.09	0.05	0.06
FGOALS-s2	FGO	cmip5	train	0.11	0.21	0.09	0.14
FIO-ESM-2-0	FIO	cmip6	train	0.1	0.19	0.07	0.11
FIO-ESM	FIO	cmip5	train	0.06	0.11	0.06	0.1
GFDL-CM4	GFD	cmip6	test	0.12	0.23	0.12	0.23
GFDL-ESM4	GFD	cmip6	test	0.14	0.27	0.15	0.26
GFDL-CM3	GFD	cmip5	test	0.24	0.47	0.21	0.38
GFDL-ESM2G	GFD	cmip5	test	0.17	0.33	0.12	0.18
GFDL-ESM2M	GFD	cmip5	test	0.11	0.2	0.1	0.19
GISS-E2-1-G	GIS	cmip6	train	0.09	0.17	0.06	0.08
GISS-E2-1-H	GIS	cmip6	train	0.08	0.14	0.06	0.08
GISS-E2-H	GIS	cmip5	train	0.05	0.1	0.05	0.07
GISS-E2-H-CC	GIS	cmip5	train	0.08	0.17	0.09	0.09

GISS-E2-R	GIS	cmip5	train	0.09	0.16	0.1	0.12
GISS-E2-R-CC	GIS	cmip5	train	0.04	0.08	0.08	0.09
HadGEM3-GC31-LL	Had	cmip6	test	0.14	0.26	0.05	0.08
HadGEM3-GC31-MM	Had	cmip6	test	0.14	0.27	0.07	0.09
HadGEM2-AO	Had	cmip5	test	0.17	0.32	0.08	0.14
HadGEM2-CC	Had	cmip5	test	0.16	0.33	0.11	0.14
HadGEM2-ES	Had	cmip5	test	0.17	0.33	0.11	0.15
INM-CM4-8	INM	cmip6	train	0.12	0.24	0.1	0.15
INM-CM5-0	INM	cmip6	train	0.09	0.17	0.07	0.12
IPSL-CM6A-LR	IPS	cmip6	test	0.15	0.3	0.08	0.11
IPSL-CM5A-LR	IPS	cmip5	test	0.1	0.19	0.08	0.1
IPSL-CM5A-MR	IPS	cmip5	test	0.08	0.14	0.09	0.13
IPSL-CM5B-LR	IPS	cmip5	test	0.16	0.29	0.12	0.13
MCM-UA-1-0	MCM	cmip6	test	0.18	0.35	0.12	0.22
MIROC-ES2L	MIR	cmip6	train	0.09	0.16	0.06	0.08
MIROC6	MIR	cmip6	train	0.1	0.17	0.05	0.06
MIROC-ESM	MIR	cmip5	train	0.12	0.22	0.06	0.09
MIROC-ESM-CHEM	MIR	cmip5	train	0.08	0.13	0.04	0.04
MIROC5	MIR	cmip5	train	0.07	0.13	0.06	0.07
MPI-ESM-1-2-HAM	MPI	cmip6	train	0.14	0.25	0.08	0.1
MPI-ESM1-2-HR	MPI	cmip6	train	0.16	0.32	0.08	0.1
MPI-ESM1-2-LR	MPI	cmip6	train	0.1	0.17	0.08	0.08
MPI-ESM-LR	MPI	cmip5	train	0.1	0.18	0.08	0.09
MPI-ESM-MR	MPI	cmip5	train	0.08	0.15	0.07	0.09
MPI-ESM-P	MPI	cmip5	train	0.1	0.19	0.07	0.08
MRI-ESM2-0	MRI	cmip6	train	0.09	0.17	0.06	0.07
MRI-CGCM3	MRI	cmip5	train	0.09	0.17	0.08	0.11
NESM3	NES	cmip6	-	0.14	0.27	0.1	0.18
NorCPM1	Nor	cmip6	test	0.08	0.16	0.06	0.08
NorESM2-LM	Nor	cmip6	test	0.15	0.28	0.06	0.07
NorESM2-MM	Nor	cmip6	test	0.12	0.24	0.09	0.1
NorESM1-M	Nor	cmip5	test	0.08	0.15	0.07	0.09
NorESM1-ME	Nor	cmip5	test	0.07	0.13	0.07	0.08
SAM0-UNICON	SAM	cmip6	-	0.07	0.12	0.06	0.09
TaiESM1	Tai	cmip6	-	0.08	0.16	0.06	0.1
UKESM1-0-LL	UKE	cmip6	-	0.13	0.25	0.07	0.1
BNU-ESM	BNU	cmip5	-	0.07	0.13	0.06	0.1
CCSM4	CCS	cmip5	train	0.08	0.15	0.07	0.11
CSIRO-Mk3-6-0	CSI	cmip5	train	0.1	0.19	0.08	0.14
inmcm4	inm	cmip5	-	0.07	0.13	0.06	0.1

[a] Model abbreviation indicates the models that stem from the same model variant and are used in the training step to partition the training samples by model.
